# Oral Bacteria and Intestinal Dysbiosis in Colorectal Cancer

**DOI:** 10.3390/ijms20174146

**Published:** 2019-08-25

**Authors:** Ioannis Koliarakis, Ippokratis Messaritakis, Taxiarchis Konstantinos Nikolouzakis, George Hamilos, John Souglakos, John Tsiaoussis

**Affiliations:** 1 Laboratory of Anatomy-Histology-Embryology, Medical School, University of Crete, 71110 Heraklion, Greece; 2 Laboratory of Translational Oncology, School of Medicine, University of Crete, 71110 Heraklion, Greece; 3 Department of Clinical Microbiology and Microbial Pathogenesis, University Hospital of Heraklion, 71110 Heraklion, Greece; 4 Department of Medical Oncology, University General Hospital of Heraklion, 71110 Heraklion, Greece

**Keywords:** colorectal cancer, oral microbiota, colonic microbiota, dysbiosis, biofilms, tumorigenesis, *Fusobacterium*, virulence factors, bacterial metabolism, chronic inflammation

## Abstract

The human organism coexists with its microbiota in a symbiotic relationship. These polymicrobial communities are involved in many crucial functions, such as immunity, protection against pathogens, and metabolism of dietary compounds, thus maintaining homeostasis. The oral cavity and the colon, although distant anatomic regions, are both highly colonized by distinct microbiotas. However, studies indicate that oral bacteria are able to disseminate into the colon. This is mostly evident in conditions such as periodontitis, where specific bacteria, namely *Fusobacterium nucrelatum* and *Porphyromonas gingivalis* project a pathogenic profile. In the colon these bacteria can alter the composition of the residual microbiota, in the context of complex biofilms, resulting in intestinal dysbiosis. This orally-driven disruption promotes aberrant immune and inflammatory responses, eventually leading to colorectal cancer (CRC) tumorigenesis. Understanding the exact mechanisms of these interactions will yield future opportunities regarding prevention and treatment of CRC.

## 1. Introduction

The human colon harbors a complex ecosystem of numerous microorganisms, including bacteria, viruses, fungi, and protozoa, referred to as the intestinal microbiota. The composition of the intestinal microbiota begins to shape since our embryonic life at birth and progressively acquires a stable functioning form, being susceptible to environmental factors that could substantially alter its structure [1,2]. The microbial genome contains consists of approximately 100 times more genes than the human genome, enabling the commensal microbiome to metabolize compounds indigestible by humans, coexisting in a synergistic relationship with the host [3]. However, under certain conditions some microbes transform into opportunistic pathogens. Although the exact triggers of this interaction are yet to be investigated, it is widely believed that the composition and function of the colonic microbiota can be affected by several factors, such as epidemiology, immune response, diet, lifestyle, alterations in the colonic microenvironment, such as the acquisition of new commensals, and drug uptake, leading to disruption of host-microbiota homeostasis.

Colorectal cancer (CRC) is one the most frequent types of cancer in both men and women [4]. The majority of cases are due to sporadic cancers (85–95%) that could be influenced by various environmental factors, and only few cases are either hereditary or related to specific predisposing diseases, such as patients with inflammatory bowel disease. Therefore, the composition and metabolism of the colonic microbiota could play an essential role in CRC pathogenesis [5].

Evidence of colonization of CRC tissue samples by members of the oral microbiota generates a hypothesis of their potential involvement in CRC tumorigenesis and various studies have revealed the crucial role of microbiota in tumorigenesis. However, the complexity of the microbial interactions and the symbiotic relationship with the host creates the need for further investigation regarding the responsible underlying mechanisms. In this review, we summarize the properties of the oral microbiota in association with intestinal dysbiosis and CRC carcinogenesis.

## 2. Oral Microbiota: An Overview

### 2.1. The Composition of the Oral Microbiota

The oral microbiota refers to the microbial communities of the human oral cavity [6]. The major heterogeneity of the residual bacteria (over 700 different species), and the proximity to numerous anatomic regions, characterize the oral microbiota as one of the most divergent and abundant microbiomes of the human body, secondary to the colonic microbiota [7,8]. Defining the oral microbiota’s composition is crucial in understanding its role in health and disease, since it constitutes a major player in maintaining oral homeostasis [6].

The oral microbiota resides in every oral tissue, including hard (teeth) and soft (buccal mucosa, tongue, soft and hard palate, gingiva) surfaces, as well as in their interfaces (supragingival and subgingival margins). Furthermore, the microbiomes of the proximal anatomic regions of the pharynx (nasopharynx, oropharynx, tonsils), the ear (middle ear, Eustachian tube) and the upper respiratory passages (nasal cavity, paranasal sinuses, larynx, trachea), display great relevance with the oral microbiota, in the context of similar bacterial composition [8]. The oral cavity and the associated structures provide the ideal conditions for bacterial growth. This is mainly achieved through a reasonably stable temperature (35–37 °C) and pH value (6.5–7.5) with minor fluctuations, providing an ideal environment to most microbial species [8,9]. Moreover, the saliva and the gingival crevicular fluid, constantly moisten these sites, thus hydrating the oral microbiota, promoting nutrient supply, and facilitating the circulation of immune and microbial factors, including antibodies and adherence molecules [8,9].

The members of the oral microbiota mainly coexist and thrive by forming complex polymicrobial communities, the “biofilms”. In this symbiotic state of co-aggregation, the various bacterial species, either aerobes or anaerobes, maintain the homeostasis of the oral ecosystem, being resistant to environmental stimuli, creating a balance between pathogens and commensals, thus aiding their survival [10]. The inter-communication of these species, developed as a result of their co-evolution, assembles large structures known as dental plaque [11]. Any alteration in the above conditions caused by either internal (e.g., genetics) or external (e.g., diet, toxicants, antibiotics) factors could enhance the pathogenetic potential of the oral microbiota, furthering the progression of oral diseases.

The oral microbiota consists of Firmicutes (mainly *Streptococcus*), Bacteroidetes (mainly *Prevotella*), and Proteobacteria, with Fusobacteria, Actinobacteria, and Haemophilus being the dominant phyla as determined by the Human Microbiome Project conducted by the National Institute of Health (NIH) [12,13]. Some bacterial species are more broadly distributed in the oral regions, such as *Fusobacterium, Gemmella, Veillonella, Streptococcus,* and *Granulicatella*, whereas other species, including *Bacteroides, Pasteurella, Prevotella, Neisseria,* and *Corynebacterium* inhabit specific oral regions [12]. For example, the oropharyngeal microbiota includes unique species, such as *Streptococcus pneumonia, Streptococcus pyogenes, Haemophilus influenzae,* and *Haemophilus parainfluenzae*. These species are not detected in other oral sites, due to the construction of the oropharyngeal epithelium which is characterized by goblet cells and a cilia coating, constituting a common respiratory and digestive passage [14]. The oral sites containing the most diverse microbiomes are the supragingival or subgingival surfaces, and the saliva [12,13]. The former regions present the most stable conditions, since they are rarely shed by the saliva or the tongue, enabling the formation of the dental plaques, which are mostly composed by early colonizers such as *Streptococcus* and *Actinomyces*, as well as later colonizers including *Veillonella, Fusobacterium, Peptostreptococcus*, and *Porphyromonas* species [15]. Regarding saliva, although it contains an unstable microbiota, displaying rapid alterations and poor nutritional content, its high diversity is primarily owed by the shedding of the bacterial communities from the various oral anatomic structures [16]. Nevertheless, the most densely populated niche in the oral cavity is the tongue, which greatly affects the total oral microbiome, since it serves as a reservoir from which the bacteria disseminate by the saliva flow, colonizing other sites of the oral cavity [17].

It is well known that the oral microbiota presents higher alpha-diversity compared to other sites, such as the skin or vaginal microbiota, however, it displays the lowest beta-diversity than other body sites. This actually pertains to fewer alterations in the oral microbiota composition between unrelated subjects [12]. In addition, it is reported that these bacterial communities share great commonalities among various individuals [18]. Such minor intra- and inter-subject differences imply that the members of the oral microbiota could serve as possible biomarkers in malignancies, such as CRC.

### 2.2. Oral Microbiota Effects in Health and Disease

The mutual commensal oral microbiota plays a crucial role in promoting not only oral, but also systemic health. Similarly, the commensal microbes in the gut microbiota are of major importance in developing the gut epithelial barrier as well as stimulating the local and systemic immunity. Mucosal IgA are not produced, and lymphoid follicles cannot be formed in the absence of microbiota [19]. The physiologic status of the oral microbiota results in colonization resistance, preventing the growth of pathogens, since the majority of available binding sites are already occupied by commensal bacteria [20]. Disruption of this balance, for example by administration of antibiotics, could elicit infections caused by opportunistic pathogens, including *Staphylococcus aureus* and *Candida* spp. [21]. Another interesting function of the oral microbiome is associated with nitrate metabolism. Through the entero-salivary circulation, approximately 25% of ingested nitrate returns to the oral cavity, which is then metabolized to nitrite by the oral microbiota. Subsequent uptake of the nitrite into the bloodstream through gastric absorption results to its conversion to nitric oxide, a significant factor of vascular physiology, which presents an anti-hypertensive action [22].

It is broadly known that the composition of the oral microbiota is changed in pathologic oral conditions; however, whether these alterations occur prior to or after disease constitutes a debated topic, which is yet to be answered. In periodontitis, for instance, microbes forming the biofilms of supragingival dental plaques are able to spread into the gingival sulcus and further into the periodontal pockets, mostly in susceptible individuals. The anaerobic environment of such tissues facilitates the growth of pathogenic bacteria, such as *Porphyromonas gingivalis, Treponema denticola* and *Tannerella forsythia*, inducing inflammatory responses [7]. A different microbial profile of the gingiva and periodontium in these patients compared to healthy subjects is considered to be a possible causative factor [15]. The importance of the oral microbiota in promoting oral disease became evident through studies in germ-free mice, where the absence of microbiota inhibited the development of periodontal disease [23]. Apart from oral diseases, the oral microbiota has also been implicated in various extra-oral conditions. There is strong evidence of an association between oral microbiota and rheumatoid arthritis [24]. More specifically, the detection of bacterial DNA in the synovial fluid of such patients indicates a possible spreading of microbes from the periodontal site to the synovium, and also periodontal therapy improved the prognosis of rheumatoid arthritis [25,26]. Individuals with periodontitis are more prone to develop cardiovascular disease [27]. This could be either a direct result of colonization of atheromatous plaques by members of oral microbiota, or an indirect effect of dental-plaque-associated induction of aberrant immunity and release of cytokines and other mediators [28]. Moreover, oral bacteria have been implicated in ventilator-assisted pneumonia, as well as in cystic fibrosis [29,30], and have been characterized to be causative agents of hepatic or brain abscesses [31] and endocarditis [32]. Periodontitis has been related to dementia and other mentally impaired diseases [33]. Interestingly, oral bacteria *P. gingivalis* and *Aggregatibacter actinomycetemcomitans* have been linked to the development of digestive cancers such as primary pancreatic adenocarcinoma [34], with species like *Fusobacterium nucleatum* presenting great invasive properties and a positive relationship with tumorigenesis.

## 3. The Concept of Intestinal Dysbiosis in Colorectal Cancer (CRC)

The human intestinal microbiota consists of over 1000 various bacterial species, mainly belonging to Firmicutes and Bacteroidetes phyla, containing beneficial and pathogenic microbes. In healthy subjects, the gut exists in homeostasis, a state that is maintained through a constant cross-talk between the residual microbiota and the host as well as within the members of microbiota, thus preventing the overgrowth of pathogens [35]. This interaction between the host and the microbiota is mutual. The intestinal microbiota simulates an “organ”-like community, performing crucial functions for our body, including biometabolism of bile acids, vitamin and amino acid synthesis, utilization of dietary compounds, vitamin production, development of immunity, and supporting the integrity of the intestinal barrier [36]. In return, the intestinal bacteria flourish in an environment full of energy sources including proteins and carbohydrates. Recently, many studies focus on the role of intestinal microbiota in the pathogenesis of CRC, by analyzing its composition and metabolome [37].

However, when alterations in the bacterial composition occur, this balance shifts in favor of pathogens that are normally suppressed by beneficial members of the intestinal microbiota, which leads to increased gut vulnerability to several pathogenic hazards, and unfavorable host effects. This disturbance of the microbiota ecosystem is termed “dysbiosis” [38]. Dysbiosis can be furthered distinguished into three separate categories, which often occur simultaneously: a) depletion of commensal bacteria, b) overgrowth of opportunistic pathogens potentially harmful microorganisms, and c) reduction in total microbiota diversity [39]. In fact, dysbiosis reflects the microbioal shifts in microbiota composition, the dysmetabolism, and the altered bacterial distribution, which negatively affect the equilibrium initiating tumorigenic phenomena [40]. Studies in animal and human models reveal that dysbiosis is associated with various disorders such as obesity, diabetes, inflammatory bowel disease, allergies, autism, colorectal adenomas and CRC [41]. Similarly, several oral disorders including gingivitis, periodontitis, caries, tonsillitis, and oral cancer, in addition to the aforementioned systemic diseases, are associated with dysbiosis of the oral microbiota [42].

The development of CRC was initially related to individual bacteria including *Helicobacter pylori*, *Streptococcus gallolyticus*, and *Escherichia coli* [43]. Nevertheless, the idea of dysbiosis points towards the involvement of numerous microbes in CRC. Many studies have analyzed and compared the intestinal microbiota between CRC patients and tumor-free subjects utilizing next-generation sequencing (NGS) technologies (e.g., 16S rRNA pyrosequencing analysis) in fecal samples [41,44]. Fecal sampling is mostly preferred, since it represents the bacterial profile of the intestinal epithelium similarly to tissue samples without the need for biopsy [45]. Current research demonstrates alterations of the intestinal microbiota between healthy subjects and patients with adenomas or CRC, suggesting a continuous shifting pattern of the microbiota during the disease progression [46]. Indeed, increased numbers of *Acinetobacter*, *Helicobacter*, and *Pseudomonas*, with higher bacterial abundance have been reported in patients with rectal adenomas compared to control subjects, implying a disruption of the intestinal balance through microbial pathogenic mechanisms, such as the alteration of intestinal luminal pH by *Helicobacter* [47]. In tissue samples from the intestinal mucosa of patients with CRC presented decreased numbers of *Blautia*, *Bifidobacterium*, and *Faecalibacterium* and higher numbers of *Fusobacterium*, *Peptostreptococcus*, *Porphyromonas*, and *Mogibacterium spp*. [41,44]. Diminution of *Clostridium* cluster IV and XIV, opposite to proliferation of *Anaerotruncus*, *Campylobacter*, *Collinsella*, *Enterococcaceae*, *Erysipelotrichaceae*, *Fusobacterium*, and *Peptostreptococcus* have been reported in fecal samples from CRC patients compared to control subjects [41,44]. These findings implicate that the initiation of the disease, in this case CRC, may be caused by modification of the balanced interaction between the host and the microbiome through the adoption of a pro-inflammatory profile by the intestinal microbiota [37]. Indeed, the populations of beneficial species that aid in the preservation of intestinal microbiota homeostasis by producing butyrate, including *Bifidobacteria*, *Roseburia*, and *Faecalibacterium prausnitzii* are decreased in CRC [48]. Simultaneously, various opportunistic pathogens, able to induce inflammatory or metabolic disorders, including *Campylobacter*, *Enterococcaceae*, *Erysipelotrichaceae*, and *Fusobacterium* are increased in CRC patients [49]. Although the enriched bacterial species are specifically localized to the tumor, the great similarities between the microbiota of the tumor area and the adjacent tumor-free mucosa, in addition to the increase in bacterial genes related to virulence factors in the tumor microenvironment, suggest that in dysbiosis the microbiota actively participates in the CRC tumorigenesis via a systemic change which impacts the whole microbial community [50]. Nevertheless, it should be noted that this causality is still based on hypotheses and is not easily proven by these observational studies.

Despite the exposure of the dysbiotic profile of the intestinal microbiota in CRC, these studies do not fully clarify the specific mechanisms that elicit the carcinogenic potential of the microbiota in CRC progression; neither have they provided adequate evidence of whether the intestinal dysbiosis acts causatively nor consequently in CRC pathogenesis. In order to further explore the role of microbiota in the onset of CRC, a dynamic model of interaction between members of the microbiota, the “bacterial driver–passenger” hypothesis, was proposed by Tjalsma et al. [51]. This model suggests that specific bacterial species with pro-tumorigenic properties (drivers) trigger the CRC development by inducing damage in the DNA of the intestinal epithelial cells. Subsequently, the disturbance in the intestinal microenvironment leads to a diminution in beneficial bacteria and colonization of the mucosa by opportunistic pathogens (passengers). Passenger bacteria are poor habitants of healthy gut, yet they utilize nutrients and other factors, including reactive oxygen species, in the tumor microenvironment presenting a competitive advantage, thus resulting in pro-inflammatory response and direct epithelial damage. On the other hand, drivers apart from their role in DNA damage they also participate in epithelium proliferation and apoptosis. The combined effects of drivers and passengers modulate the dysbiosis of microbiota regarding CRC.

Intestinal dysbiosis not only is involved in the tumorigenesis but also determines the treatment efficacy. The metabolic capacity of the colonic microbiota regarding anti-tumoral compounds, and its ability to regulate host’s immunity and inflammatory response is linked to the therapeutic outcome [52]. These effects combined indicate the crucial involvement of host’s intestinal microbiota in modulating the efficacy of chemotherapeutic and immunotherapeutic agents [53]. Regarding chemotherapy, the dysbiotic intestinal microbiota as a result of antibiotic therapy reduces the tumor response to oxaliplatin treatment, due to depletion of microbiota-derived reactive oxygen species (ROS) production and, thus, reduction of tumoric cell apoptosis [54]. On the other hand, the introduction of probiotics (namely *Lactobacilli* or *Enterococci*) restores the physiological composition of the intestinal microbiota and stimulates the T helper 17 (Th17) immune response, thus improving the efficacy of cyclophosphamide treatment [55,56]. With the current evidence suggesting a cross-talk between immunity and the tumor, immunotherapy is highlighted as a promising type of cancer treatment, indicating the intestinal microbiota as a novel target for therapy [57]. Indeed, it is reported that the integrity of the commensal intestinal microbiota greatly affects the optimal responses to cancer immunotherapy via regulation of myeloid-derived cell function in the tumor microenvironment [58]. It has been demonstrated that immunotherapy with ipilimumab, an antibody against cytotoxic T-lymphocyte- associated antigen-4 (CTLA-4), disrupts the function of regulatory T-cells (Treg) and increases the abundance of *Bacteroides fragilis*, thus improving the efficacy of this treatment [59]. Furthermore, *Bifidobacterium* stimulates the activation of dendritic cells and optimizes the response to antibody treatment against programmed cell death protein ligand 1 (PD-L1) [60]. Nevertheless, the above findings are based on mouse model studies and human clinical trials will verify their validity for future clinical application. Further human clinical trials will verify the validity of the above findings for future clinical application.

## 4. Oral Bacteria and Intestinal Dysbiosis

Many studies indicate that members of the oral microbiota are involved in intestinal dysbiosis, indirectly affecting the composition of the intestinal microbiota via dissemination into the gut. *P. gingivalis* has been extensively associated with intestinal dysbiosis in view of periodontitis causing systemic diseases, since it greatly influences the oral immunity and induces oral dysbiosis [61,62].

In a study by Arimatsu et al. [63], *P. gingivalis* was orally provided to C57BL/6N mice twice a week for a total of 5 weeks. The administration resulted in endotoxemia, and diminished ileal gene expression of tight junction proteins, such as Zonula Occludens-1 (ZO-1). Analysis using 16S rRNA pyrosequencing demonstrated an alteration in intestinal microbiota composition with increased abundance of Bacteroidales (mainly *Paraprevotella* and *Barnesiella*). Although *P. gingivalis* belongs to Bacteroidales order such bacterial-specific DNA was not detected in blood. Periodontitis and its related microbiota could result in increased blood endotoxin levels, yet intestinal dysbiosis induced by gut translocation of oral bacteria could be a causative factor.

In a former study by the same authors, C57BL/6N mice were orally administered with *P. gingivalis* in a single dose [64]. The intestinal microbiota differed significantly from sham-treated mice, with decreased Firmicutes and elevated Bacteroidetes phyla. At the genus level, unclassified S24–7 and *Prevotella* increased, whereas Clostridiales decreased. Similar dysbiotic profile has been detected in colitis [65]. Moreover, downreguation in gene expression of the intestinal tight-junction proteins Tjp1 and Ocln indicated disruption of the intestinal barrier, which was mainly attributed to endotoxemia. Also, the presence of less than 0.01% of *P. gingivalis* in the fecal samples triggers its pathogenic potential impairing the host-microbiota balance [66].

In another study by Sato et al. [67], the oral administration of *P. gingivalis*, and *Prevotella intermedia* in DBA/1J mice with experimentally collagen-induced arthritis (CIA), led to endotoxemia, systemic inflammation, disruption of intestinal barrier, and intestinal dysbiosis. However, these results were specific for *P. gingivalis*, resulting in decreased abundance of Bacteroidetes (mainly *Bacteroides* and *Prevotella*) as opposed to increased Firmicutes (*Allobaculum*). *P. gingivalis* also stimulated Th17 immune response in Peyer’s patches and mesenteric lymph nodes and increased serum levels of interleukin (IL)-17. Thus, the activation of gut intestinal immunity and dysbiosis led to aggravation of arthritis.

In order to test whether oral periodontopathic bacteria could interfere with host metabolism, *P. gingivalis* W83 was administered orally in C57BL/6 mice twice weekly for a 5-week period [68]. Pyrosequencing analysis demonstrated altered intestinal microbiota composition, with increased *Ruminococcus* and decreased *Dorea* species compared to sham-treated mice. Metabolomics revealed enhanced biosynthesis of several amino acids, such as alanine, glutamine, histidine, tyrosine, and phenylalanine. These findings were associated with the development of obesity and insulin resistance [69].

Other members of the oral bacteria have also been reported to be involved in the development of systemic diseases in association with intestinal dysbiosis. A study conducted in human subjects with liver cirrhosis, a disease related to intestinal dysbiosis [70], demonstrated by using metagenomics and gene catalogues that the majority (54%) of the patient-enriched, taxonomically assigned members of intestinal microbiota originated from the oral cavity [71]. These bacteria (13 species in total) mainly belonged to *Veillonella* and *Streptococcus*, followed by *Fusobacterium*, *Aggregatibacter*, and *Megasphaera*. The authors suggested a massive invasion of the gut from oral commensals, with a positive correlation of the populations of the invading bacteria with the disease severity. Bile dysmetabolism due to cirrhosis could possibly impair the intestinal barrier, rendering the gut more sensitive to colonization with extra-gut bacteria [72].

Since periodontitis has been related to chronic liver conditions such as non-alcoholic fatty liver disease (NAFLD) [73], with the intestinal microbiota interfering in NAFLD pathogenesis [74], the periodontopathogen *A. actinomycetemcomitans* was orally administered to C57BL/6J mice in either normal chow (NCAa, NCco) or high-fat diet (HFAa, HFco) for a 6-week period [75]. The Aa groups showed greater insulin resistance with less glucose tolerance compared to co groups, and both HF groups presented higher hepatic steatosis. Furthermore, the ingestion of *A. actinomycetemcomitans* induced perturbations in intestinal microbiota, decreasing the abundance of *Turicibacter*. Since the expression of inflammatory genes did not significantly differ between Aa and co groups, it was proposed that the glucose metabolism was altered due to induction of an orally-driven intestinal dysbiosis with diminution of butyrate-producing species [76].

Recently, Lourenco et al. [77] analyzed the intestinal microbiota in fecal samples from subjects with oral diseases, being either gingivitis (*n* = 14) or chronic periodontitis (*n* = 23). The composition of the intestinal microbiota demonstrated less alpha-diversity, with elevated abundance of Firmicutes, Euryarcheota, Proteobacteria, and Verrucomicrobiota as well as decreased Bacteroidetes, compared to healthy controls. The decrease in diversity was contrary to previous animal studies [63,64], indicating an intestinal dysbiosis related to disease. Several oral pathogens were detected in the fecal samples from all groups, including *Dialister*, *Eubacterium*, *Filifactor*, *Fusobacterium*, *Parvimonas*, *Porphyromonas*, *Prevotella*, *Tannerella*, and *Treponema*. Furthermore, increased populations of oral taxa, such as *Campylobacter rectus*, *Dialister invisus*, *Filifactor alocis*, *Fusobacterium* spp., *Leptotrichia* spp., *Oribacterium* spp., *Porphyromonas edodontalis*, *Parvimonas micra*, *Prevotella* spp., *Slenomonas* spp., *Tannerella* spp., and *Treponema* spp., in the intestinal microbiota were associated with periodontal inflammation and loss of attachment. How these oral pathobionts reached the intestinal mucosa, inducing dysbiosis and inflammation was not examined.

The importance of periodontitis and related bacteria in the modulation of gut dysbiosis became profound after periodontal therapy in patients with cirrhosis [78]. This led to improvement in intestinal dysbiosis, with increased commensal bacteria (Ruminococcaceae and Lachnospiraceae) and decreased opportunistic pathogens (Enterobacteriaceae) in addition to reduced taxa of oral origin (Porphyromonadaceae and Streptococcaceae) in fecal samples in cirrhotic patients, especially in those with hepatic encephalopathy. Systemic inflammatory markers, such as IL-6, IL-1β, white blood cell (WBC) count, and endotoxin levels were also reduced following periodontal therapy.

The above animal and human studies indicate that the colonic microbiota may be affected by oral bacteria, such as *P. gingivalis*, leading to dysbiosis. In particular, long-term oral ingestion of *P. gingivalis*, similarly to periodontitis, may influence intestinal dybiosis. Apart from *P. gingivalis*, other periodontopathogens including *A. actinomycetemcomitans*, can also disseminate to the colon. This oral–colon link may constitute another route for oral bacteria-mediated systemic inflammatory responses.

## 5. Oral Bacteria Detected in CRC

Several studies have recently examined and validated the presence of various bacterial members of the oral microbiota in gastrointestinal tumors, especially regarding CRC [79].

Nakatsu et al. [80] characterized the intestinal microbial communities in patients with adenoma, CRC, or healthy individuals. Detection of abundant bacteria of oral origin, including *Fusobacterium*, *Gemella*, *Peptostreptococcus* and *Parvimonas* indicated a dynamic symbiotic metacommunity presenting a strong relationship with CRC tumorigenesis. Significant correlations of bacterial taxa in adenoma, and co-exclusive relationships that persisted in CRC revealed that these oral bacteria were involved in a dysbiotic state of intestinal microbiota, which probably occurred during the cancerous progression.

Subsequent studies in fecal samples from patients with colonic adenomas, also revealed elevated numbers of oral genera, including *Actinomyces*, *Corynebacterium*, *Haemophilus*, *Mogibacterium*, and *Porphyromonas*, compared to controls [81]. Increased abundance of periodontal pathogens such as *Fusobacterium*, *Oscillibacter*, *Peptostreptococcus*, *Porphyromonas*, *Roseburia*, and *Ruminococcus* were also observed in fecal samples from patients with CRC [45,82].

In an innovative study, Flemer et al. [83] analyzed the microbiota in oral swabs, fecal samples, and colonic mucosa of patients with CRC, colonic polyps, or healthy individuals, using 16S rRNA gene sequencing. They revealed that several strains were similar between oral swabs and fecal samples, involving microbes that contribute to the formation of oral biofilms as late colonizers, including *Fusobacterium nucleatum*, *Peptostreptococcus stomatitis*, and *Parvimonas micra*. Although these species were enhanced in CRC, they were also present in control subjects. The colonization of the colonic mucosa by oral bacteria was negatively associated with increased populations of Lachnospiraceae, such as *Anaerostipes*, *Blautia* and *Roseburia*, implying a beneficial role of such members in preventing the development of CRC. This effect could be mediated through a healthy diet, since the richness of the above protective bacteria was negatively associated with a Western-type diet which is known to be connected to CRC carcinogenesis [83]. Thus the oral microbiota was distinctive and predictive, suggesting a potential tool for CRC screening.

The predictive role of the oral microbiota in CRC pathogenesis was further supported by a large retrospective study by Momen-Heravi et al. [84]. The authors detected that individuals with chronic periodontitis, especially with severe tooth loss (<17 teeth), were high-risk for CRC development with poorer prognosis. These findings were associated mostly with proximal tumors, compared to distal or rectal, since polymicrobial communities are more associated with proximal sites of CRC [85].

The risk of developing CRC in relation to oral microbiota was recently investigated in a large cohort study [86]. Analysis of mouth rinse samples via 16S rRNA gene sequencing demonstrated that oral pathogenic taxa such as *Treponema denticola*, Bifidobacteriaceae, and *Prevotella* (*P. denticola, P. intermedia, P. oral taxon 300*) were positively associated with increased risk of CRC, whereas Carnobacteriaceae, Erysipelotrichaceae, *Prevotella melaninogenica*, *Streptococcus*, and *Solobacterium* indicated a reduced risk for CRC development.

Regarding CRC, although many oral bacteria are involved, *Fusobacterium nucleatum* is a paramount species that is regularly identified in fecal as well as in mucosal samples from CRC patients [49,87,88]. *F. nucleatum* is a well-known pro-inflammatory, invasive, anaerobic, oral pathogen, with evident association with dental plaque and periodontitis as a late colonizer [89]. However, *F. nucleatum* usually co-exists with other members of oral microbiota, such as *Porphyromonas* spp. (mainly *P. asaccharolytica* and *P. gingivalis*) which consist some of the most typically increased taxa in CRC individuals [44,90,91]. It is also well-established that *F. nucleatum* and *P. gingivalis* synergistically initiate oral tumorigenesis [91].

One of the earlier studies concerning the role of Fusobacteria in CRC, demonstrated enriched sequences of *Fusobacterium* spp. (*F. nucleatum, F. mortiferum*, and *F. necrophorum*) in CRC tissue using quantitative polymerase chain reaction (PCR), 16S rDNA sequencing, and fluorescent in situ hybridization (FISH) in CRC tissue, while bacteria belonging to Firmicutes and Bacteroidetes phyla were decreased [87]. Castellarin et al. [49] speculated an overabundance of *Fusobacterium* (especially *F. nucleatum*) in CRC tissue compared to controls, which was positively correlated with lymph node metastasis. In another study, 16S rRNA gene sequencing in fecal samples revealed reduced overall diversity of the intestinal microbiota, with elevated numbers of *Fusobacterium* spp. and *Porphyromonas* spp. and reduced abundance of *Clostridium* spp., between fecal samples from CRC patients and controls [90]. To investigate whether *F. nucleatum* could have a predictive or prognostic significance, quantitative PCR was conducted in tissue and fecal samples from patients with CRC [88]. *F. nucleatum* was over-represented in cancerous tissue compared to corresponding normal tissue. Interestingly, longer overall survival time was observed in CRC patients with low levels of *F. nucleatum* than in subjects with either moderate or high levels of this species. Similarly, in another study the prognostic value of *F. nucleatum* was evaluated in tissue samples from 1102 CRC patients, where the detection of *F. nucleatum* DNA was linked to poorer prognosis in CRC cases [92]. Thus, *F. nucleatum* could possibly serve as a non-invasive biomarker for CRC screening. Since CRC is related to specific genetic or epigenetic mutations alterations, Tahara et al. [93] hypothesized that Fusobacteria could associate with molecular features of CRC. Quantitative PCR in tissue samples from CRC patients demonstrated enrichment of *Fusobacterium* spp. compared to adjacent tumor-free tissue or healthy subjects. These results were positively associated with CpG island methylator phenotype (CIMP) status, human mutL homolog 1 (hMLH1) methylation, tumor protein 53 (TP53) wild type, chromodomain helicase DNA binding protein (CHD)7/8 mutation, and microsatellite instability (MSI). These correlations of *Fusobacterium* spp. with these molecular subsets support the pathogenic role of this bacterium in CRC development. Gao et al. [94], aimed to investigate the intestinal dysbiosis in CRC by comparing data from 16S rRNA gene sequencing in tumor tissue and adjacent disease-free mucosa in CRC samples from proximal or distal colonic sites. *Fusobacterium* and *Lactococcus* were increased, whereas *Escherichia-Shigella* and *Pseudomonas* were reduced in tumor tissue compared to adjacent healthy tissue. This could imply that impairment in the tumor microenvironment and the formation of more anaerobic situations, facilitating the growth of opportunistic pathogens of oral origin such as *Fusobacterium*. Additionally, enrichment of the oral species *Prevotella* in proximal CRC has been linked to enhanced IL-17 producing cells in colonic mucosa of CRC patients [95]. Thus, oral pathogens thrive in intestinal dysbiosis, dynamically interacting with other microbiota members, promoting CRC tumorigenesis.

Considering that the microbiota composition differs between proximal (cecum to transverse colon) and distal (splenic flexure to sigmoid colon) colonic segments, Mima et al. [92] tested whether the proportion of *F. nucleatum* follows a similar distribution pattern across colon. The populations of *F. nucleatum* demonstrated a gradual enrichment from rectal (2%) to cecal (11%) highly-abundant colonic tumors. These data were in accordance with previous studies that reported higher numbers of *F. nucleatum* in proximally located CRC [96]. Notably, the cecum demonstrates the highest risk of CRC occurrence per mucosal surface area [97], and cecal tumors show great prevalence of *KRAS* mutations [98].

Since *F. nucleatum* is increased in CRC patients, some studies tried to implement this finding in clinical practice, such as CRC screening and therapy. The overgrowth of *F. nucleatum* in CRC tissue samples associated with *KRAS* mutation, tumor size and correlated with reduced overall survival times, leading to the development of a DNA test, highly sensitive for this bacterium, for prognostic and screening purposes in Japanese population [99]. It was also reported that *F. nucleatum* demonstrated increased abundance in tissue samples from patients with recurrent CRC after chemotherapy [100]. It became evident that *F. nucleatum* regulated a complex molecular network of signaling pathways of innate immunity (toll-like receptor 4 and MyD88), specific micro-RNAs, and autophagy, modulating chemoresistance of CRC. Thus, it was proposed that anti-bacterial therapy, exclusively targeting *F. nucleatum*, could act synergistically with chemotherapy to improve clinical outcomes in CRC.

Another recent study by Komiya et al. [101], an analysis of *F. nucleatum* strains in tumor tissue and saliva from patients with CRC, revealed great similarity of the bacterial composition between these specimens, with almost 40% of patients presenting identical strains of *F. nucleatum*. This indicates that *F. nucleatum* in the intestinal microbiota in CRC subjects originates from the oral cavity, supporting the aforementioned studies and strengthening the hypothesis about an orally driven intestinal dysbiosis in CRC.

The results of the above studies regarding the detection of oral bacteria in CRC are summarized in Table 1**.**

## 6. Possible Mechanisms of Oral Microbiota Involvement in CRC Dysbiosis

### 6.1. Dissemination of Oral Bacteria into the Intestinal Environment

The extensive detection of oral bacteria in the microbiotic profile of intestinal dysbiosis related to CRC implies that colonization of the intestine by such microbes plays a key role in understanding CRC pathogenesis. Segata et al. [102] in the Human Microbiome Project reported a significant overlap between the fecal and oral microbiota, with almost 45% similarity in bacterial taxa. Thus, intestinal colonization could be mediated through translocation of oral microbes. To further examine this hypothesis, Li et al. [103] transplanted human saliva into germ-free mice developed a human oral microbiota-associated (HOMA) mouse model, via transplantation of human saliva into gnotobiotic mice. The majority of the oral genera widely distributed across the digestive tract of HOMA mice. In the colon, bacteria belonging to *Actinomyces*, *Fusobacterium*, *Haemophilus*, *Streptococcus*, *Trichococcus*, and *Veillonella* were especially abundant. Co-housing with human microbiota-associated (HMA) mice, developed from fecal transplantation to gnotobiotic mice, led to significant ecological invasion of the intestinal ecosystem by oral bacteria. This effect was prominent in the small intestine, with the genera *Empedobacter*, *Enterococcus*, *Moraxella*, *Porphyromonas*, *Streptococcus*, and *Trichococcus* being dominant. One important function of the intestinal microbiota is to defend the host against opportunistic pathogens through competing behavior of commensal bacteria by expressing antimicrobial factors, such as bacteriocins, and regulation of the mucosal immunity. These events lead to enhancement of the mucosal barrier and reduction of pathogenic translocation and colonization [104]. Moreover, bacteria of oral origin, including Actinobacteria, Fusobacteria, and Clostridia, among others, present the ability to surpass this protective barrier and thrive in the germ-free colon [105]. Similarly the disruption of the microbiotic and physical barrier resulted in the dissemination of oral bacteria across the cephalocaudal digestive axis.

While the origin of the intestinal colonizers related to disease remains elusive, concerning its endogenous (from the oral cavity) or exogenous (from the environment) acquirement, a study by Atarashi et al. [106] demonstrated that *Klebsiella* strains, derived from salivary samples of patients with inflammatory bowel disease, inducing intestinal Th1 inflammatory response in germ-free mice following colonization of the colonic mucosa. This finding suggests that the ectopic intestinal dissemination of oral bacteria constitutes a rare divergent event, and thus a hallmark of disease. To further support the above statement, the microbiota of fecal samples from healthy and diseased individuals was recently analyzed [107]. The results revealed an extensive transmission of the majority of oral bacteria into the colon, with subsequent colonization, in healthy subjects. This event was more profound in CRC patients, especially for previously described pathobionts [108], such as *Parvimonas micra*, *Peptostreptococcus stomatis*, and *F. nucleatum*. This implies that the pathogenic species in the intestinal microbiota that are associated with CRC are sourced endogenously, with the oral cavity serving as a major reservoir in shaping the intestinal microbiota.

Although the precise detailed mechanisms of bacterial transfer from the oral cavity to the colon are not clear, two possible routes have been described.

The first route of bacterial translocation that could possibly change the composition of the colonic microbiota is through continuous swallowing of oral bacteria [64]. Swallowed saliva, ingested food and fluids, shed the microbiota of the oral cavity or the oropharynx, providing a passage to the gastrointestinal tract. The human production of saliva ranges from 0.75 to 1.5 L per day, containing a vast amount of oral microbes. For instance, in chronic periodontitis, *P. gingivalis* a major periodontopathic pathogen can be swallowed in amounts ranging between 10^8^–10^10^ [109]. The oral and intestinal microbiota remain relatively distinctive by several mechanisms, including bile acids in the duodenum, and gastric acid [110,111]. Oral bacteria able to resist the harsh acidic gastric environment could sustain their viability across this route [112]. This is a particular characteristic of *P. gingivalis*, hence aiding its migration to the colon altering the composition and functional capacity of the residual microbiota [67]. In addition to this, chronic exposure to proton pump inhibitors, could further enhance the intestinal colonization of oral bacteria, shifting the gastric pH towards a less acidic value [113]. However, studies in germ-free mice reported that *P. gingivalis* was not isolated in fecal samples, suggesting inefficiency in colonizing the colonic mucosa [114]. This fact applies to other oral pathogens such as *F. nucleatum*, since quantitative PCR on tissue specimens from CRC patients showed the detection of this bacterium in only 13% of cases [92]. The need for oral gavage of *F. nucleatum* on a daily basis for initiation of tumorigenesis, and the relatively poor potential in colonizing healthy colonic mucosa [105], indicates that oral bacteria are incorporated into the intestinal microbiota via additional mechanisms. One possible factor could be the swallowing of dead bacterial components that upregulate the expression of bacterial virulence factors and induce cytotoxic phenomena, a concept known as “necrotrophy-necrovirulence”. In an in vitro study, a ratio of at least 10:1, regarding dead to living bacterial cells, was correlated with significant growth of periodontal pathogens, especially *Porphyromonas* spp. (*P. gingivalis* and *P. intermedia*) [115]. Upregulated gene expression related to virulence factors, including gingipain genes *rgpA*, *rgpB* and *kgp*, as well as collagenase *prtC* and fimbriae *fimA* genes reflected the above effects.

The second route of bacterial dissemination could be through spreading via bloodstream and systemic circulation (bacteremia) to extra-oral sites, including the joints, the heart, and the colon. It has been shown that oral microbiota can directly access bloodstream during usual dental activities, including tooth brushing, removal or mastication [116]. Nevertheless, inflammatory conditions of the oral cavity, namely periodontitis, may facilitate bacteremia, since during periodontitis the periodontal vasculature is more dilated and proliferated as a result of chronic inflammation. *F. nucleatum* and *P. gingivalis* are able to invade bloodstream through ulcerated gingival pockets [117]. A recent study by Tsukasaki et al. [118] reported that experimentally-induced periodontitis resulted in translocation of oral bacteria in the hepatic and splenic tissue of mice. Tooth extraction and amelioration of gingivitis inhibited bacterial transfer, proposing that bacteremia was caused through impairment of oral epithelial barrier. Host cells could also be utilized as “Trojan horse” for the bacterial spreading through blood [117]. According to this, oral bacteria such as *P. gingivalis* can survive inside immune cells, including dendritic cells or macrophages, subsequently disseminating to various body sites [119].

Another possibility is that the colonic inflammation and shifts in intestinal microbiota may be prerequisites for the colonization by oral pathogens. More evidence is needed in order to describe the routes of bacterial translocation of oral pathogens into the colon in detail.

### 6.2. The Role of Oral Polymicrobial Biofilms in CRC

One common characteristic of both oral and intestinal microbiota is their ability to form biofilms, complex multimicrobial communities surrounded by a polymeric matrix, which facilitate their growth, bypass the defensive mechanisms of the host, and promotes the colonization of mucosal surfaces via adhesion mediated by various glycoproteins. Biofilms in oral diseases, such as periodontitis, are usually developed in three stages [120]. The primary colonizers are *Streptococcus* spp. and *Actinomyces* spp., which reside in the subgingival surface, creating an anaerobic environment that is ideal for intermediate colonizers such as *F. nucleatum*, attracting late colonizers like *P. gingivalis*, *Tannerella forsythia*, *Treponema denticola*, and *A. actinomycetemcomitans* through expression of several adhesins [15,121].

The colonic environment could stimulate these conditions, indicating that oral microbes could inhabit the colon in similar biofilm-like structures. Indeed, recent studies confirm the detection of biofilms on colonic mucosa of CRC patients as well as healthy subjects, which also contain members of the oral microbiota. Dejea et al. [85] demonstrated, with the use of FISH and 16S rRNA gene sequencing analysis, the enhanced presence of *F. nucleatum* in biofilms deriving from adenoma and CRC specimens. Interestingly, *F. nucleatum* was absent in healthy colonic tissue and all the isolated biofilms were polymicrobial, with the matched biofilm-positive tumor and healthy tissue samples harboring invasive bacteria. Thus, biofilms associated with CRC present similar structure and pathogenic potential to those in oral diseases, possibly interacting with CRC tumorigenesis. Other studies also reveal that intestinal biofilms contain commensal (*Parvimonas*, *Peptostreptococcus*, *Prevotella*) in addition to pathogenic (*F. nucleatum*, *P. gingivalis*) periodontal bacteria, which could result in CRC development [122]. Warren et al. [123] observed the coexistence of oral anaerobic bacteria including *F. nucleatum*, *Campylobacter*, and *Leptotrichia* in paired normal and CRC tissues. These species were inter-correlated, forming a cooperative polymicrobial network in tumoric tissue with other species such as *P. gingivalis*. Moreover, isolation of *Campylobacter* strains demonstrated the ability to coaggregate with *F. nucleatum*, hence creating the hypothesis that the latter may serve as a bridging microorganism, colonizing the intestinal mucosa by attracting other compatible oral bacteria. Recently, it was reported that bacterial biofilms are typically presented in the majority of proximal (89%) compared to distal (12%) CRC [124]. Subsequent metabolomics showed a significant pro-carcinogenic potential of bacterial biofilms in colonic mucosa. This is consistent with other studies that observed bacterial biofilms containing mucus-invasive species, with overgrowth of *F. nucleatum* in proximal CRC [125].

The pathogenic effect of bacterial biofilms in CRC is mediated through disruption of the colonic mucus layer. The mucus covering the colonic epithelium is a bi-layered gel-like structure, which is formed through secretion of MUC2 by goblet cells. The inner layer is densely packed, attached on the luminal side of enterocytes via trans-membranous proteins and goblet cells, and impenetrable to bacteria [126]. As the mucin production continues, the mucus layer expands towards the intestinal lumen. The exposure to various bacterial enzymes (glycosidases, proteases) disintegrates the mucus, creating an optimal environment for microbial colonization [127]. As a result, the outer layer is formed, which is unattached and flows with the fecal content, housing commensal members of the intestinal microbiota. These mucus layers play a crucial role in colonic immunity, protecting the intestinal mucosa from protracted interaction with the microbiota. Furthermore, the mucus contains several peptides with antibacterial activity (defensins, cathelicidins) and IgA antibodies, secreted by enterocytes and submucosal immune cells, creating a defensive mechanism against invasion by pathogenic microbes [126]. The inner mucus layer in eubiosis is not inhabited by bacteria, whereas, during intestinal dysbiosis the down-regulated synthesis of MUC2 and antimicrobial peptides, due to increased bacterial pathogenicity, leads to intestinal biofilm formation. These bacterial biofilms are associated with enhanced microbial attachment and invasion into the colonic epithelium, inflammation (activation of IL-6 and signal transducer and activator of transcription 3 [STAT3] pathways), aberrant immune responses, and thus increased cytotoxicity or genotoxicity [124]. These phenomena eventually cause improper epithelial cell proliferation and colorectal tumorigenesis [85].

The “driver–passenger” model, which was previously discussed regarding intestinal dysbiosis (see. Section 3), can also be applied to the biofilm formation in periodontal diseases [117]. In the context of oral dysbiosis, driver pathogens such as *P. gingivalis* can further shape the biofilm structure, altering the growth and gene expression of passenger bacteria, and impairing host immunity, through several virulence factors [128]. However, due to the indications from the above studies, this model can be expanded including “bridging” strains like *F. nucleatum* [129]. These species can invade oral epithelial cells, by producing adhesins, surface ligands, and proteolytic enzymes. This effect is gradually enlarged creating a stable microenvironment harboring major pathogens such as *P. gingivalis* into the biofilm. Under these conditions, the various bacteria cooperate forming a self-preservation community which could initiate pro-inflammatory diseases such as periodontitis via induction of oral dysbiosis. This is known as the “polymicrobial synergy and dysbiosis” (PSD) model [129].

A similar model of dysbiosis seems to apply in the colonic ecosystem. The driver and passenger species in CRC co-exist in a complex interaction inside a bacterial biofilm in the tumor tissue, and its composition is evolving over time. Regarding *F. nucleatum*, the ongoing debate is whether it behaves as a driver or a passenger in intestinal dysbiosis. When the “driver–passenger” model was proposed, the role of oral bacteria in CRC had not been explored; hence, *F. nucleatum* was described as a bacterial “passenger”. Classic driver bacteria included species such as *Bacteroides fragilis*, *Enterococcus faecalis*, and *E. coli*, which were able to impair the epithelium and promote tumorigenesis through production of genotoxins and ROS [130]. In a study by Kostic et al. [131], members of *Fusobacterium* spp. were found abundant in colonic adenomas from human subjects, and daily administration of *F. nucleatum* in *Apc^Min/+^* murine model of CRC for 8 weeks enhanced tumor multiplicity and recruitment of tumor-infiltrating myeloid cells, forming a pro-inflammatory profile. The requirement of daily administration of *F. nucleatum* for such long time period in order to initiate tumorigenesis implies that additional species may participate in this interaction. These findings suggest that *F. nucleatum* could be labeled as a driver bacterium in this model of intestinal dysbiosis, since it promotes tumor development, furthering the colonization of the colonic environment by other pathogenic oral species which serve as passengers. Despite this speculation, some studies show contradictory results. Although the abundance of *F. nucleatum* was found to be increased in CRC patients compared to controls, there was no significant correlation in colorectal adenomas, either in fecal [132] or tissue samples [88]. In a recent study by Tomkovich et al [133] several bacterial taxa such as Bacteroidetes, Proteobacteria, and Lachnospiraceae were enriched in mucosal-related biofilms in preclinical murine models of CRC, while members of *Fusobacterium* spp. were not detected. Early establishment of tumorigenesis was possible without the presence of Fusobacteria, confirming studies which indicate that Fusobacteria are mainly involved in advanced or metastatic CRC [134]. These data are also supported by studies reporting that *F. nucleatum* does not present pro-tumorigenic or proinflammatory abilities in gnotobiotic *Apc^Min/+^* mice [135]. Only specific *F. nucleatum* possess tumorigenic properties through interaction with other species of intestinal microbiota. All these findings imply that *F. nucleatum* merely resembles a passenger rather than a driver of intestinal dysbiosis in CRC. Further investigation regarding the detailed cross-talk between oral bacteria and intestinal flora in biofilms will clarify their role as drivers or passengers in CRC dysbiosis.

### 6.3. The Metabolic Properties of Oral Bacteria in the Colon

The shift of the oral microbiota composition towards more anaerobic strains is indicative of periodontitis [117]. Apart from the alterations in oxygen demands, the microbial metabolism also changes into proteolytic and asaccharolytic [136]. As a result, ammonia and short-chain fatty acids (SCFAs) are produced in the gingival crevicular fluid (GCF) of the subgingival space, neutralizing the pH, thus enhancing the proteolytic activity of several oral species (such as *P. intermedia*) which further sustain this disruption. Finally, these events increase the abundance of pathobionts like *P. gingivalis*, *F. nucleatum, P. intermedia,* and *Campylobacter*, perpetuating the proteolytic vicious cycle regarding proteins in exfoliated epithelial cells and (GCF) [136].

Notably, the responsible metabolic pathways are not similar between species, with *F. nucleatum* and *P. intermedia* preferring smaller molecules, like amino acids, whereas *P. gingivalis* mainly disintegrates dipeptides into amino acids. Hence, the former bacteria form an optimal environment for pathogens to thrive, which in turn support their nutrition, in a continuous manner. The enhanced proteolytic ability of this bacterial consortium promotes immune responses, creating a preferable nutritional basis for biofilm development, with concurrent suppression of defensive mechanisms such as complement immunity [117]. This implies that in oral dysbiosis metabolic cooperation between oral bacteria stimulates the onset of diseases like periodontitis [128].

Apart from their synergistic metabolism, members of the oral microbiota are able to synthesize various carcinogenic substances. For example, volatile sulfur compounds (VSCs), including hydrogen sulfide (H_2_S), which are widely known for their toxic and inflammatory potential even at low concentrations, are highly produced in the oral cavity by *A. actinomycetemcomitans*, *F. nucleatum*, *P. intermedia*, and *P. gingivalis* [137].

Similar to the oral cavity, these coordinating interactions may take place in the colonic environment following colonization of oral bacteria. Colonic mucosa demonstrates an anaerobic environment, with more neutral pH than the oral cavity, which is frequently shedded off, promoting nutrition as well as adhesive sites for pathogenic bacteria [138]. During their passage from the oral cavity to the colon, several oral bacteria adopt the aforementioned anaerobic, asaccharolytic and proteolytic metabolic profile [139], enabling them to degrade the mucins and extracellular matrix in the colon, resulting in infiltration of mucus layer and invasion into the mucosa through disruption of epithelial junctions [140]. The perturbed mucosal ecosystem promotes the overgrowth of proteolytic pathogens like *Peptostreptococcus,* and *Porphyromonas*. For instance, *P.gingivalis* produces cysteine proteases called “gingipains”, being specific to either lysine (Kgp) or arginine (Rgp) [141], which are actively involved in bacterial biofilm development, with subsequent stimulation of vascular permeability and tissue impairment [139]. Moreover, gingipains are able to degrade immunological factors, including immunoglobulins like IgA, components of the complement, and cytokines, hence triggering an antibacterial immune response aiding their survival. Oral streptococci also possess the ability to cleave IgA through beta-galactosidase and neuraminidase [142]. The ongoing destruction of host proteins in the colon by oral bacteria induces a chronic inflammatory state, which continuously generates nutritional substances for microbiota, and could eventually promote CRC tumorigenesis [143].

Colonic biofilms of oral bacteria can further impair the colon by synthesizing carcinogenic metabolites, ROS and polyamines [144]. Enhanced production of polyamine metabolites spermine and diacetylspermine is a distinctive characteristic of colonic biofilms that has been associated with DNA insults in colonic epithelium, since proper antibiotic treatment decreased biofilm formation in addition to polyamine levels [145]. Furthermore, polyamines are mandatory factors for biofilm development and microbiota preservation, inducing aberrant tumorous proliferation [146]. As a result, the oral communities produce essential metabolites, supporting the integrity of their biofilms, while deteriorating intestinal metabolism and provoking tumoric proliferation [144,145]. H_2_S is an agent with genotoxic properties which could cause genomic instability or aggregated DNA mutations [147]. Up-regulated expression of numerous H_2_S-producing enzymes has been reported in CRC, such as cystathionine-β-synthase which promotes the overproduction of H_2_S, in turn affecting tumor development and spread by induction of migrating, invasive, and proliferative endocytic pathways, and stimulation of tumor angiogenesis [148].

Other substances which have been associated with increased risk of CRC, such as alcohol, are metabolized by oral bacteria into hazardous compounds. Many species of streptococci (*Streptococcus oralis, Streptococcus gordonii, Streptococcus mitis* etc.) are capable of converting alcohol to acetaldehyde, a well-known carcinogen, through metabolization by the enzyme alcohol dehydrogenase (ADH) [149]. Such ADH-producing bacteria have been detected in oral cancer and could possibly lead to colon carcinogenesis [150]. Muto et al. reported that species belonging to *Neisseria* can synthesize acetaldehyde in extreme amounts in vitro, compared to other oral bacteria, indicating their major potential in potentiating human tumorigenesis [151]. Moreover, oral microbes may also be involved in the enhanced activation of tumorigenic nitrosamines, namely nitrosodiethylamine (NDEA), from tobacco smoking [152]. Such products are indisputable carcinogens, promoting the formation of DNA adducts in vitro [153]. Tobacco also furthers the metabolism of ethanol to acetaldehyde by oral microbiota, suggesting a synergistic effect of alcohol-smoking related carcinogenesis [79].

Recent studies have detected L-tryptophane (Trp) as an important amino acid, maintaining a balanced relationship between the intestinal microbiota and host immunity. In particular, oral members of intestinal microbiota, such as *F.nucleatum*, are capable of metabolizing Trp to various derivatives (tryptamine, indole, skatole), regulating the immune response of the colonic epithelium through binding with the aryl hydrocarbon receptor (AhR) [154]. Lamas et al. showed amelioration of colonic inflammation following administration of Trp-metabolizing species, like *Lactobacillus* spp. [155], suggesting that extreme deprivation of AhR ligands could result in aberrant intestinal immunity, possibly leading to CRC [156].

The proteolytic activity of oral bacteria in the intestinal environment leads to the production of SCFAs that aggregate in subgingival space in high amounts, causing an inflammatory response that furthers the progression of oral diseases [136]. However, similar SCFA production in the colon following fermentation of dietary fibers by commensal bacteria is beneficial, mediating colonic homeostasis [157]. SCFA synthesis inhibits inflammation and apoptosis, reduces luminal pH, and sustains mucosal immunity, thus forming an unfavorable microenvironment for colonization by oral pathogens, such as *F. nucleatum* [158], and protecting from CRC progression [143]. These data indicate that dietary and lifestyle manners may be significantly correlated with intestinal colonization by *F. nucleatum* in CRC [159]. Indeed, fiber- and starch-rich diets have been related to reduced incidence of *F. nucleatum*-associated CRC, whereas the consumption of western-type diet leads to increased risk of *F. nucleatum*-positive CRC [160]. Although the anti-inflammatory activity of SCFAs could permit immune impairment by oral pathogens, the exact role of orally-mediated synthesis of SCFAs in CRC tumorigenesis has not yet been fully elucidated.

### 6.4. Virulence Factors of Oral Bacteria Inhibit Apoptosis and Modulate Inflammation and Immune Response in the Colon

As we previously mentioned several oral species such as *Fusobacterium* and *Porphyromonas* are highly detected in CRC. Although many other pathogenic or commensal members of the oral microbiome, such as the genera *Peptostreptococcus*, *Prevotella*, *Parvimonas*, and *Gemella*, have also been reported to be increased in CRC, their virulence has not been examined individually [161]. Studies of the former two pathogens have revealed numerous virulence mechanisms with anti-apoptotic and inflammatory properties [162].

Although the association of *F. nucleatum* with CRC is widely known, the responsible mechanistic pathways of this interaction remain elusive. Most studies focus on the role of Fap2 and FadA, two proteins of the outer membrane of *F. nucleatum*. The invasive potential of *F. nucleatum* is mediated through the adhesive Fap2 peptide [163]. Fap2 inhibits the stimulation of lymphocytes and cytotoxic natural killer (NK) cells when bound to the inhibitory “T-cell immunoglobulin and immunoreceptor tyrosine-based inhibitory motif domain” (TIGIT) receptor of these cells, creating an immunosuppressive and thus protective microenvironment for tumors infected with *F. nucleatum* from host immunity [164]. In a study by Abed et al [165], it was found that *F. nucleatum* achieves enrichment in the colorectal tumor through binding of Fap2 to the polysaccharide D-galactose-β(1–3)-*N*-acetyl-d-galactosamine (Gal-GalNAc), which is greatly expressed in CRC, revealing Gal-GalNAc as a possible therapeutic target in tumors infected with *F. nucleatum* [165]. Attachment and subsequent invasion into the colonic epithelial cells in CRC is mediated by the adhesion of FadA, a protein unique to *F. nucleatum*, to E-cadherin [166]. This binding also initiates the expression of several oncogenic and inflammatory genes as well as the Wnt pathway. More specifically, internalization through clathrin leads to the activation of Wnt cascade enabling CRC tumorigenesis [91]. Despite the profound role of FadA and Fap2 in promoting inflammatory and carcinogenic phenomena, Tomkovich et al. recently demonstrated that the presence of these *F. nucleatum*-specific proteins is not adequate to elicit such responses in murinary CRC mouse model [135].

*P. gingivalis* presents anti-apoptotic activity via activation of many different signaling pathways. In the gingival epithelium, the surface purinergic receptor P2X7 presents pro-apoptotic ability following binding to adenosine triphosphate (ATP). Cleavage of ATP by an enzyme secreted by *P. gingivalis*, the nucleoside diphosphate kinase (NDK), inhibits cell apoptosis promoting tumorigenesis [167]. P2X7 receptor is also present in other tissues, including colonic epithelium, playing an essential role in regulating innate and adaptive colonic immunity in addition to cell proliferation, although its behavior in inflammatory and cancerous diseases, such as CRC, is still ambiguous [168]. *P. gingivalis* also promotes the anti-apoptotic cascade involving Janus kinase 1 (Jak1), protein kinase B (Atk), and STAT3, which regulates intrinsic mitochondrial apoptosis pathways [169]. Activation of phosphoinositide 3-kinase (PI3K) by Jak1 enables the phosphorylation of the Bcl-2-associated death promoter (Bad) and caspase-9 inhibiting their pro-apoptotic properties [170]. This interaction leads to upregulation of the anti-apoptotic Bcl-2 and downregulation of pro-apoptotic (Bcl-2)-associated X (Bax) protein, a fact that became evident in the gingival epithelium [171]. *P. gingivalis* also enables cell proliferation affecting the S-phase of cell cycle via downregulation of apoptotic p53 by regulation of cyclin/cyclin-dependent kinase (CDK) activity [162]. Furthermore, *P. gingivalis* secretes unique molecules, called gingipains that promotes the nuclear factor (NF)-κΒ pathway after binding to protease activated receptor (PAR), subsequently activating metalloproteinase-9 (MMP-9) through cleavage of its pro-enzyme. MMP-9 furthers tumor cell invasion and migration as a result of degradation of basal membrane composition [172].

Colonic immunity consists of a vast variety of components, such as immune and epithelial cells along with their products (cytokines, growth factors), antibacterial factors and other supporting cells and mediators. Local and systemic immunity is also regulated by recognition of several components of colonic microbiota including microbe-associated molecular patterns (MAMPs). MAMPs commonly include substances like lipopolysaccharide (LPS), and others such as peptidoglycan, bacterial DNA or RNA, polysaccharides, and flagella. Receptors responsible for this are called pattern recognition receptors (PRRs) and are divided into numerous families, such as the Toll-like receptors (TLRs), the nucleotide-binding oligomerization (NOD)-like receptors (NLRs), the absent in melanoma 2 (AIM2)-like receptors, the RIG-I-like receptors, the OAS-like receptor and C-type lectin receptors [173]. TLRs are one of the most important receptors, that are generally expressed in immune cells (dendritic cells and macrophages), and are able to induce colonic epithelial growth, and sustain the integrity of the mucosal barrier, as well as producing several crucial factors for maintaining colonic homeostasis including chemokines, secretory IgA, mucus, and antibacterial peptides [174]. When microorganisms invade the colonic barrier, MAMPs and other bacterial products are recognized by the PRRs on the cells of host immunity, subsequently promoting pro-inflammatory response, accompanied by secretion of chemokines and cytokines, finally differentiating the immune response [175].

TLR4 in particular is an essential receptor for LPS recognition, which may further tumor progression since it is highly expressed in CRC [176]. *F. nucleatum* and its corresponding LPS can bind to this receptor, activating the P-PAK1 signaling and beta-catenin pathway [177]. It has also been reported that CRC cells form mouse models infected by *F. nucleatum* present increased stimulation of TLR4 that enhances the expression of microRNA-21 (miR-21) ultimately increasing tumor proliferation [177]. Upregulation of several inflammatory cytokines with tumorigenic potential, such as IL-6, IL-8, tumor necrosis factor-alpha (TNF-α), and cyclooxygenase-2 (Cox-2), has been detected in many studies using either in vitro cultures or immunoassay techniques in CRC tissue samples [131,178].

*F. nucleatum*-enriched CRCs demonstrated increased release of C-C motif chemokine ligand 20 (CCL20), stimulation of NF-κΒ signaling, and induction of tumor infiltration through migration of activated macrophages [179,180]. *F. nucleatum* has been linked to immune suppression, through promotion of lymphocytic apoptosis [180]. The abundance of *F. nucleatum* has been found to be inversely proportional to CD3+ T-cell density [181], although other studies failed to reveal a significant relationship between these two elements [180]. This association of immunosuppressive phenomena with bacterial dysbiosis in cancer has also been supported by the detection of dysbiotic intestinal microbiota in patients with primary immunodeficiency, such as X-linked inhibitor of apoptosis (XIAP) deficiency. Interestingly, not only do the intestinal microbiota present alterations in its composition in these patients, but also some of the taxa with increased abundance (*Scardovia*, *Fusobacterium*, *Rothia dentocariosa*, and *Veillonella*) are members of the oral microbiota [182] that are also involved in the pathogenesis of inflammatory bowel disease and CRC [183]. Thus, the intestinal microbiota of patients with primary immunodeficiency presents distinct perturbations, indicating a primary defect in host immunity as a core of intestinal dysbiosis.

Concerning *P. gingivalis*, the production of NDK induces ATP-mediated mitochondrial and cytosolic ROS, which play a key role in upregulation of transcription factors related to inflammation and tumorigenesis [184], in addition to stimulation of antioxidant glutathione response via interaction between P2X7 receptor and nicotinamide adenine dinucleotide phosphate (NADPH)-oxidase [185]. Other members of the oral microbiota, especially *Peptostreptococcus*, *Parvimonas* and *Prevotella*, are also able to promote an inflammatory response, disrupting the function of epithelial and endothelial cells, impairing the coposition of the extracellular matrix, and affecting local levels of numerous cytokines such as IL-1β, IL-6, IL-17, IL-23, TNF-α, and matrix metalloproteinases MMP-8 and MMP-9 [186].

All in all, the orally-driven intestinal dysbiosis in favor of opportunistic pathobionts results in impairment of the colon mucosa, increased bacterial invasion and translocation, stimulating the innate and adaptive immunity, leading to a chronic inflammatory state [187]. More specifically, the activated components of the innate immunity (dendritic cells, macrophages, and NK cells) secrete pro-inflammatory cytokines, including IL-12, IL-23, TNF-α, and interferon-gamma (IFN-γ), which in turn induces the response of the components of the adaptive immunity (T and B lymphocytes) [36]. The major result of this inflammatory response is the upregulation of specific epithelial signaling cascades, including NF-κΒ and STAT3 [188], and the production of reactive nitrogen and oxygen species. These phenomena leads to oxidative stress, DNA insult, irregular cell proliferation, and, finally, the development of colorectal adenomas and cancerogenesis.

**Figure 1:**
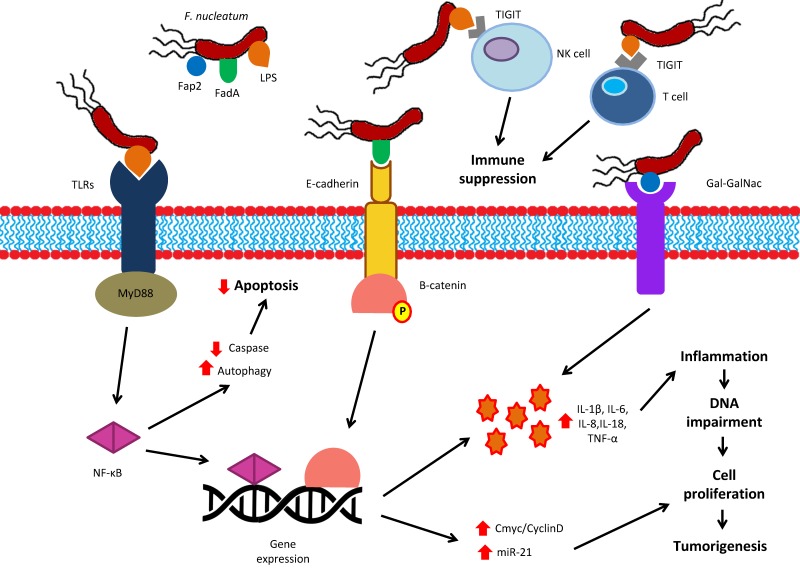
Schematic summary of the molecular pathways of *F. nucleatum* in CRC tumorigenesis. *F. nucleatum* mediates its oncogenic properties through three major components: the Fap2, and FadA molecules along with the LPS. LPS can interact with TLRs (namely TLR2 or TLR4), activating the MyD88 and NF-κΒ pathway. This interaction leads to reduced caspase activity and increased autophagy, resulting in reduced apoptosis. Furthermore, FadA binds to E-cadherin, causing dephosphorylation and activation of β-catenin. NF-κΒ and β-catenin alter the gene expression, increasing the synthesis of pro-inflammatory cytokines (IL-1β, IL-6, IL-8, IL-18, TNF-α) and upregulating oncogenic pathways of Cmyc/CyclinD and miR-21. The pro-inflammatory state is further enhanced by the binding of Fap2 to Gal-GalNAc. Additionally, the interaction of LPS with the TIGIT receptor of NK and T cells leads to suppression of anti-tumor immunity. Eventually, these events create inflammation which impairs DNA, promotes cell proliferation and results in CRC tumorigenesis. CRC: colorectal cancer; Gal-GalNAc: D-galactose-β(1–3)-N-acetyl-D-galactosamine; IL: interleukin, LPS: lipopolysaccharide; miR: microRNA: NF-κΒ: nuclear factor kappa-beta; NK: natural-killer; TIGIT: T-cell immunoglobulin and immunoreceptor tyrosine-based inhibitory motif domain; TLR: toll-like receptor; TNF-α: tumor necrosis factor-alpha. Upward red arrows: enhancement/stimulation; Downward red arrows: reduction.

The various molecular pathways of tumorigenesis associated with *F. nucleatum* are represented in Figure 1.

## 7. Conclusions

**Figure 2:**
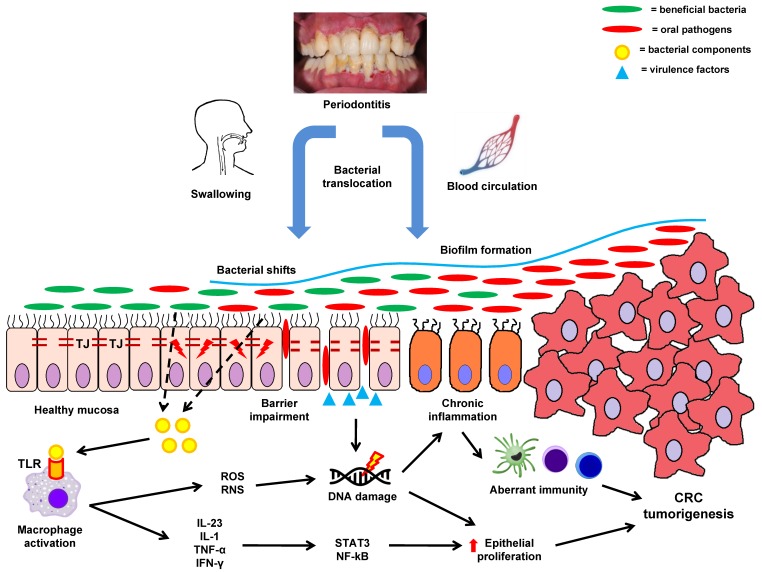
A proposed model of orally-driven intestinal dysbiosis in CRC development. In periodontitis several oral pathogens, such as *Fusobacterium nucleatum* or *Porphyromonas gingivalis*, are abundant in oral biofilms. This situation aids the dissemination of oral pathogens into the colon via either swallowing of the saliva or bloodstream. In the colonic environment they are incorporated into the intestinal microbiota. As a result, bacterial shifts and the production of virulence factors create a microbiotic instability and outgrowth of pathogens leading to intestinal dysbioisis. Subsequent biofilm formation and secretion of several bacterial components (e.g., metabolites, toxins, MAMPs), lead to macrophage activation and disruption of the mucosal barrier. Pro-inflammatory cytokines, such as IL-23, IL-1, TNF-α, and IFN-γ, enable signaling pathways through STAT3 and NF-κΒ activation, enhancing the proliferation of epithelial cells. Moreover, the release of ROS and RNS and the increased exposure of submucosal environment by virulaence factors and other bacterial products or toxins result in DNA damage and mutations. These events promote a chronic inflammatory state which stimulates an aberrant immune response and further impairs the colonic epithelium. Inevitably, the result of the above interactions is the initiation and progression of CRC carcinogenesis. CRC: colorectal cancer; IFN-γ: interferon-gamma; IL: interleukin; MAMPs: microbe associated molecular patterns; NF-κΒ: nuclear factor kappa-beta; RNS: reactive nitrogen species; ROS: reactive oxygen species; STAT3: signal transducer and activator of transcription 3; TJ: tight junctions; TNF-α: tumor necrosis factor-alpha. Upward red arrow: enhancement.

The current understanding of microbiome, metabolome, metagenome and other omics in various studies help us to clarify the involvement of the oral microbiota in CRC carcinogenesis (Figure 2). A unified inspection of the microbial community in CRC indicates that metabolic activity and composition of a multispecies bacterial community seem to define the core of carcinogenesis in relation to intestinal dysbiosis. Hence, a consortium of inflammatory responses, virulence factors and impaired epithelial signaling in the context of a polymicrobial oral biofilm with synergistic properties creats a suitable microenvironment for the development of disrupted and irregular interactions between the host and microbiota. Oral periodontopathic bacteria can further translocate into the colorectum becoming a part of a potentially pathogenic microbiota with altered composition. Using mechanisms equivalent to those in the oral cavity, they synthesize growth and virulence factors, progressively eradicating the benefical bacteria. This inevitably creates instability of the commensal microbiota and favors the superiority of orally-derived opportunistic pathogens resulting in intestinal dysbiosis. Mucosal adhesion and biofilm formation, accompanied by increased concentration of toxic metabolites and enhanced proteolytic activity, can disrupt the integrity of the colonic barrier. All the above mechanisms, combined with aberrant immunity, can result in inflammation and CRC tumorigenesis.

Future studies should focus on clarifying the detailed mechanisms that determine CRC carcinogenesis through an orally-driven intestinal dysbiosis, especially regarding the exact role of *F. nucleatum* virulence proteins. Additionally, the virulence factors and pathogenic pathways of oral bacteria, other than *F. nucleatum*, in combination with biofilm formation and metabolic activity should also be investigated, furthering our knowledge about their involvement in intestinal dysbiosis. Nevertheless, whether the intestinal colonization by these bacteria requires a dysbiosis of oral microbiota or an already established dysbiotic bacterial community in the colon is still not clear, but cannot be ignored. Since the majority of oral bacteria in CRC dysbiosis are periodontopathic bacteria, good oral hygiene, periodontal treatment, and probiotics may aid the prevention of intestinal diseases that are mediated by oral bacteria. One limitation of the literature is that the studies published to date regarding the role of the oral microbiota in CRC have been inconsistent, with the exception of findings about *F. nucleatum*, possibly due to the small sample sizes. Studies with larger numbers of samples are needed for more solid results. Since the oral bacteria are actively participating in the intestinal dysbiosis and the induction of tumor proliferation, treatments that target the microbiota in order to modulate immune response reveal a novel route for cancer inmmunotherapy. Translating the promising findings from immunotherapy studies into substantial clinical treatments requires a deep understanding of the complex mechanisms of the aforementioned biological pathways. Hence, elucidation of the immune and inflammatory mechanisms of microbiota-mediates CRC pathogenesis will yield new opportunities for CRC prevention, prognosis, as well as treatment strategies.

## Figures and Tables

**Table 1:** Summary of various experimental studies regarding the presence of oral bacteria in colorectal cancer (CRC).

Oral Bacteria.	Sampling/Size	Methods	Main Findings	References
*Fusobacterium*, *Gemella*, *Peptostreptococcus* and *Parvimonas*	—Colonic mucosa/ control (*n* = 61), colonic adenoma-normal adjacent pair (*n* = 47), tumor tissue-normal adjacent pair (*n* = 52)	16S rRNA gene sequencing	—Increased abundance of presented bacteria in CRC —Mucosal microbiota demonstrates distinct changes across stages of CRC tumorigenesis.	Nakatsu et al. 2015 [80]
*Actinomyces*, *Corynebacterium*, *Haemophilus*, *Mogibacterium*, and *Porphyromonas*	—Feces/colonic adenoma patients (*n* = 233), control (*n* = 547)	16S rRNA gene sequencing	—Increased abundance of presented bacteria in colonic adenomas	Hale et al. 2017 [81]
*Fusobacterium*, *Oscillibacter*, *Peptostreptococcus*, *Porphyromonas*, *Roseburia*, and *Ruminococcus*	—Colonic mucosa/tumor tissue (*n* = 59), colonic adenoma (*n* = 21), control (*n* = 56)	16S rRNA gene sequencing, real-time qPCR	—Increased abundance of presented bacteria in CRC	Flemer et al. 2017 [45]
*Fusobacterium nucleatum*, *Peptostreptococcus stomatitis*, and *Parvimonas micra*	—Oral swabs, feces, colonic mucosa/CRC patients (*n* = 99), colonic adenoma patients (*n* = 32), Controls (*n* = 103)	16S rRNA gene sequencing	—Increased abundance of presented bacteria in CRC —Oral microbiota is distinctive and predictive in CRC	Flemer et al. 2018 [83]
*Treponema denticola*, Bifidobacteriaceae, and *Prevotella* Carnobacteriaceae, Erysipelotrichaceae, *Prevotella melaninogenica*, *Streptococcus*, and *Solobacterium*	—Mouth rinse/CRC patients (*n* = 231), Control (*n* = 462)	16S rRNA gene sequencing	—The former group of bacteria was associated with increased risk of CRC —The latter group of bacteria was associated with reduced risk of CRC	Yang et al. 2018 [86]
*Fusobacterium* spp. (*F. nucleatum, F. mortiferum*, and *F. necrophorum*)	—Colonic mucosa/tumor tissue-normal adjacent pair (*n* = 95)	qPCR, 16S rRNA gene sequencing, FISH	—Bacteria belonging to Fusobacterium were abundant in CRC	Kostic et al. 2012 [87]
*Fusobacterium (F. nucleatum)*	—Colonic mucosa/ tumor tissue-matched normal tissue (*n* = 99)	qPCR, 16S rRNA gene sequencing	—Increased abundance of Fusobacterium in CRC was positively associated with lymph node metastasis	Castellarin et al. 2012 [49]
*Fusobacterium* spp., *Porphyromonas* spp.	—Feces/CRC patients (*n* = 47), control (*n* = 94)	16S rRNA gene sequencing	—Increased abundance of presented bacteria in CRC patients —Decreased abundance of *Clostridium* spp. was simultaneously detected	Ahn et al. 2013 [90]
*Fusobacterium nucleatum*	—Colonic mucosa/tumor tissue-matched normal tissue (*n* = 122), colonic adenoma-matched normal tissue (*n* = 52) —Feces/CRC patients (*n* = 7), colonic adenoma patients (*n* = 24), controls (*n* = 25)	qPCR	—Patients with high levels of *F. nucleatum* presented a significantly shorter survival time that patients with low levels of this species	Flanagan et al. 2014 [88]
*Fusobacterium* spp.	Colonic mucosa/tumor tissue (*n* = 149), normal adjacent tissue (*n* = 89), control (*n* = 72)	qPCR	—Fusobacterium enhancement is associated with specific molecular subsets of CRC	Tahara et al. 2014 [93]
*Fusobacterium* spp. and *Lactococcus* spp.	—Colonic mucosa/ tumor tissue (*n* = 31), normal adjacent tissue (*n* = 20)	16S rRNA gene sequencing	—Increased abundance of presented bacteria in CRC —*Pseudomonas* and *Escherichia-Shigella* were decreased	Gao et al. 2015 [94]
*Fusobacterium nucleatum*	—Colonic mucosa/tumor tissue (*n* = 1102)	qPCR	—Increased abundance of this species in proximal CRC	Mima et al. 2016 [92]
*Fusobacterium nucleatum*	—Colonic mucosa/tumor tissue (*n* = 100), normal tissue (*n* = 72)	Droplet digital PCR	—Overabundance of this species correlated with KRAS mutation, tumor size, and shorter survival time	Yamaoka et al. 2018 [99]
*Fusobacterium nucleatum*	—Colonic mucosa/tumor tissue (*n* = 296)	HT RNA sequencing, real time qPCR	—*Fusobacterium nucleatum* promotes chemoresistance through modulation of autophagy in CRC	Yu et al. 2017 [100]
*Fusobacterium nucleatum*	—Colonic mucosa, saliva/CRC patients (*n* = 14)	AP-PCR, 16S rRNA gene sequencing	—Similar strains of *Fusobacterium nucleatum* are presented between oral cavity and colon in CRC patients	Komiya et al. 2019 [101]

AP-PCR: arbitrarily primed polymerase chain reaction, CRC: colorectal cancer, HT: high-throughput, qPCR: quantitative PCR, FISH: fluorescent in situ hybridization.

## References

[1] Aagaard K., Ma J., Antony K.M., Ganu R., Petrosino J., Versalovic J. (2014). The placenta harbors a unique microbiome. Sci. Transl. Med..

[2] Maffei V.J., Kim S., Blanchard E.t., Luo M., Jazwinski S.M., Taylor C.M., Welsh D.A. (2017). Biological Aging and the Human Gut Microbiota. J. Gerontol. A Biol. Sci. Med. Sci..

[3] Qin J., Li R., Raes J., Arumugam M., Burgdorf K.S., Manichanh C., Nielsen T., Pons N., Levenez F., Yamada T. (2010). A human gut microbial gene catalogue established by metagenomic sequencing. Nature.

[4] Ferlay J., Soerjomataram I., Dikshit R., Eser S., Mathers C., Rebelo M., Parkin D.M., Forman D., Bray F. (2015). Cancer incidence and mortality worldwide: Sources, methods and major patterns in GLOBOCAN 2012. Int. J. Cancer.

[5] Arthur J.C., Jobin C. (2011). The struggle within: Microbial influences on colorectal cancer. Inflamm. Bowel. Dis..

[6] Dewhirst F.E., Chen T., Izard J., Paster B.J., Tanner A.C., Yu W.H., Lakshmanan A., Wade W.G. (2010). The human oral microbiome. J. Bacteriol..

[7] Wade W.G. (2013). The oral microbiome in health and disease. Pharmacol. Res..

[8] Aas J.A., Paster B.J., Stokes L.N., Olsen I., Dewhirst F.E. (2005). Defining the normal bacterial flora of the oral cavity. J. Clin. Microbiol..

[9] Marcotte H., Lavoie M.C. (1998). Oral microbial ecology and the role of salivary immunoglobulin A. Microbiol. Mol. Biol. Rev..

[10] Dowd S.E., Wolcott R.D., Sun Y., McKeehan T., Smith E., Rhoads D. (2008). Polymicrobial nature of chronic diabetic foot ulcer biofilm infections determined using bacterial tag encoded FLX amplicon pyrosequencing (bTEFAP). PLoS ONE.

[11] Kolenbrander P.E., Andersen R.N., Blehert D.S., Egland P.G., Foster J.S., Palmer R.J. (2002). Communication among oral bacteria. Microbiol. Mol. Biol. Rev..

[12] Huse S.M., Ye Y., Zhou Y., Fodor A.A. (2012). A core human microbiome as viewed through 16S rRNA sequence clusters. PLoS ONE.

[13] Zhou Y., Gao H., Mihindukulasuriya K.A., La Rosa P.S., Wylie K.M., Vishnivetskaya T., Podar M., Warner B., Tarr P.I., Nelson D.E. (2013). Biogeography of the ecosystems of the healthy human body. Genome Biol..

[14] Hull M.W., Chow A.W. (2007). Indigenous microflora and innate immunity of the head and neck. Infect. Dis. Clin. North Am..

[15] Teles R., Teles F., Frias-Lopez J., Paster B., Haffajee A. (2013). Lessons learned and unlearned in periodontal microbiology. Periodontol 2000.

[16] Mager D.L., Haffajee A.D., Devlin P.M., Norris C.M., Posner M.R., Goodson J.M. (2005). The salivary microbiota as a diagnostic indicator of oral cancer: A descriptive, non-randomized study of cancer-free and oral squamous cell carcinoma subjects. J. Transl. Med..

[17] Danser M.M., Gomez S.M., Van der Weijden G.A. (2003). Tongue coating and tongue brushing: A literature review. Int. J. Dent. Hyg..

[18] Zaura E., Nicu E.A., Krom B.P., Keijser B.J. (2014). Acquiring and maintaining a normal oral microbiome: Current perspective. Front. Cell. Infect. Microbiol..

[19] Hooper L.V., Littman D.R., Macpherson A.J. (2012). Interactions between the microbiota and the immune system. Science.

[20] Vollaard E.J., Clasener H.A. (1994). Colonization resistance. Antimicrob. Agents Chemother..

[21] Sullivan A., Edlund C., Nord C.E. (2001). Effect of antimicrobial agents on the ecological balance of human microflora. Lancet Infect. Dis..

[22] Kapil V., Milsom A.B., Okorie M., Maleki-Toyserkani S., Akram F., Rehman F., Arghandawi S., Pearl V., Benjamin N., Loukogeorgakis S. (2010). Inorganic nitrate supplementation lowers blood pressure in humans: Role for nitrite-derived NO. Hypertension.

[23] Tlaskalova-Hogenova H., Stepankova R., Hudcovic T., Tuckova L., Cukrowska B., Lodinova-Zadnikova R., Kozakova H., Rossmann P., Bartova J., Sokol D. (2004). Commensal bacteria (normal microflora), mucosal immunity and chronic inflammatory and autoimmune diseases. Immunol. Lett..

[24] Rutger Persson G. (2012). Rheumatoid arthritis and periodontitis-inflammatory and infectious connections. Review of the literature. J. Oral Microbiol..

[25] Temoin S., Chakaki A., Askari A., El-Halaby A., Fitzgerald S., Marcus R.E., Han Y.W., Bissada N.F. (2012). Identification of oral bacterial DNA in synovial fluid of patients with arthritis with native and failed prosthetic joints. J. Clin. Rheumatol..

[26] Kaur S., Bright R., Proudman S.M., Bartold P.M. (2014). Does periodontal treatment influence clinical and biochemical measures for rheumatoid arthritis? A systematic review and meta-analysis. Semin. Arthritis Rheum..

[27] Beck J.D., Offenbacher S. (2005). Systemic effects of periodontitis: Epidemiology of periodontal disease and cardiovascular disease. J. Periodontol..

[28] Serra e Silva Filho W., Casarin R.C., Nicolela E.L., Passos H.M., Sallum A.W., Gonçalves R.B. (2014). Microbial diversity similarities in periodontal pockets and atheromatous plaques of cardiovascular disease patients. PLoS ONE.

[29] Heo S.M., Haase E.M., Lesse A.J., Gill S.R., Scannapieco F.A. (2008). Genetic relationships between respiratory pathogens isolated from dental plaque and bronchoalveolar lavage fluid from patients in the intensive care unit undergoing mechanical ventilation. Clin. Infect. Dis..

[30] Filkins L.M., Hampton T.H., Gifford A.H., Gross M.J., Hogan D.A., Sogin M.L., Morrison H.G., Paster B.J., O’Toole G.A. (2012). Prevalence of streptococci and increased polymicrobial diversity associated with cystic fibrosis patient stability. J. Bacteriol..

[31] Antunes A.A., de Santana Santos T., de Carvalho R.W., Avelar R.L., Pereira C.U., Pereira J.C. (2011). Brain abscess of odontogenic origin. J. Craniofac. Surg..

[32] Pierce D., Calkins B.C., Thornton K. (2012). Infectious endocarditis: Diagnosis and treatment. Am. Fam. Physician.

[33] Gil-Montoya J.A., Sanchez-Lara I., Carnero-Pardo C., Fornieles F., Montes J., Vilchez R., Burgos J.S., Gonzalez-Moles M.A., Barrios R., Bravo M. (2015). Is periodontitis a risk factor for cognitive impairment and dementia? A case-control study. J. Periodontol..

[34] Fan X., Alekseyenko A.V., Wu J., Peters B.A. (2018). Human oral microbiome and prospective risk for pancreatic cancer: A population-based nested case-control study. Gut.

[35] Tremaroli V., Backhed F. (2012). Functional interactions between the gut microbiota and host metabolism. Nature.

[36] Keku T.O., Dulal S., Deveaux A., Jovov B., Han X. (2015). The gastrointestinal microbiota and colorectal cancer. Am. J. Physiol. Gastrointest. Liver Physiol..

[37] Candela M., Turroni S., Biagi E., Carbonero F., Rampelli S., Fiorentini C., Brigidi P. (2014). Inflammation and colorectal cancer, when microbiota-host mutualism breaks. World J. Gastroenterol..

[38] Bien J., Palagani V., Bozko P. (2013). The intestinal microbiota dysbiosis and Clostridium difficile infection: Is there a relationship with inflammatory bowel disease?. Therap. Adv. Gastroenterol..

[39] DeGruttola A.K., Low D., Mizoguchi A., Mizoguchi E. (2016). Current Understanding of Dysbiosis in Disease in Human and Animal Models. Inflamm. Bowel. Dis..

[40] Abreu M.T., Peek R.M. (2014). Gastrointestinal malignancy and the microbiome. Gastroenterology.

[41] Wu N., Yang X., Zhang R., Li J., Xiao X., Hu Y., Chen Y., Yang F., Lu N., Wang Z. (2013). Dysbiosis signature of fecal microbiota in colorectal cancer patients. Microb. Ecol..

[42] Proctor D.M., Relman D.A. (2017). The Landscape Ecology and Microbiota of the Human Nose, Mouth, and Throat. Cell Host Microbe.

[43] Arthur J.C., Perez-Chanona E., Muhlbauer M., Tomkovich S., Uronis J.M., Fan T.J., Campbell B.J., Abujamel T., Dogan B., Rogers A.B. (2012). Intestinal inflammation targets cancer-inducing activity of the microbiota. Science.

[44] Chen W., Liu F., Ling Z., Tong X., Xiang C. (2012). Human intestinal lumen and mucosa-associated microbiota in patients with colorectal cancer. PLoS ONE.

[45] Flemer B., Lynch D.B., Brown J.M., Jeffery I.B., Ryan F.J., Claesson M.J., O’Riordain M., Shanahan F., O’Toole P.W. (2017). Tumour-associated and non-tumour-associated microbiota in colorectal cancer. Gut.

[46] Peters B.A., Wu J., Hayes R.B., Ahn J. (2017). The oral fungal mycobiome: Characteristics and relation to periodontitis in a pilot study. BMC Microbiol.

[47] Sanapareddy N., Legge R.M., Jovov B., McCoy A., Burcal L., Araujo-Perez F., Randall T.A., Galanko J., Benson A., Sandler R.S. (2012). Increased rectal microbial richness is associated with the presence of colorectal adenomas in humans. Isme. J..

[48] Fukuda M., Komiyama Y., Mitsuyama K., Andoh A., Aoyama T., Matsumoto Y., Kanauchi O. (2011). Prebiotic treatment reduced preneoplastic lesions through the downregulation of toll like receptor 4 in a chemo-induced carcinogenic model. J. Clin. Biochem. Nutr..

[49] Castellarin M., Warren R.L., Freeman J.D., Dreolini L., Krzywinski M., Strauss J., Barnes R., Watson P., Allen-Vercoe E., Moore R.A. (2012). Fusobacterium nucleatum infection is prevalent in human colorectal carcinoma. Genome Res..

[50] Lu Y., Chen J., Zheng J., Hu G., Wang J., Huang C., Lou L., Wang X., Zeng Y. (2016). Mucosal adherent bacterial dysbiosis in patients with colorectal adenomas. Sci. Rep..

[51] Tjalsma H., Boleij A., Marchesi J.R., Dutilh B.E. (2012). A bacterial driver-passenger model for colorectal cancer: Beyond the usual suspects. Nat. Rev. Microbiol..

[52] Schwabe R.F., Jobin C. (2013). The microbiome and cancer. Nat. Rev. Cancer.

[53] Gopalakrishnan V., Helmink B.A., Spencer C.N., Reuben A., Wargo J.A. (2018). The Influence of the Gut Microbiome on Cancer, Immunity, and Cancer Immunotherapy. Cancer Cell.

[54] Iida N., Dzutsev A., Stewart C.A., Smith L., Bouladoux N., Weingarten R.A., Molina D.A., Salcedo R., Back T., Cramer S. (2013). Commensal bacteria control cancer response to therapy by modulating the tumor microenvironment. Science.

[55] Viaud S., Saccheri F., Mignot G., Yamazaki T., Daillere R., Hannani D., Enot D.P., Pfirschke C., Engblom C., Pittet M.J. (2013). The intestinal microbiota modulates the anticancer immune effects of cyclophosphamide. Science.

[56] Daillere R., Vetizou M., Waldschmitt N., Yamazaki T., Isnard C., Poirier-Colame V., Duong C.P.M., Flament C., Lepage P., Roberti M.P. (2016). *Enterococcus hirae* and *Barnesiella intestinihominis* Facilitate Cyclophosphamide-Induced Therapeutic Immunomodulatory Effects. Immunity.

[57] Koliarakis I., Psaroulaki A., Nikolouzakis T.K., Kokkinakis M., Sgantzos M.N., Goulielmos G., Androutsopoulos V.P., Tsatsakis A., Tsiaoussis J. (2018). Intestinal microbiota and colorectal cancer: A new aspect of research. JBUON..

[58] Hibberd A.A., Lyra A., Ouwehand A.C., Rolny P., Lindegren H., Cedgard L., Wettergren Y. (2017). Intestinal microbiota is altered in patients with colon cancer and modified by probiotic intervention. BMJ. Open Gastroenterol..

[59] Vetizou M., Pitt J.M., Daillere R., Lepage P., Waldschmitt N., Flament C., Rusakiewicz S., Routy B., Roberti M.P., Duong C.P. (2015). Anticancer immunotherapy by CTLA-4 blockade relies on the gut microbiota. Science.

[60] Sivan A., Corrales L., Hubert N., Williams J.B., Aquino-Michaels K., Earley Z.M., Benyamin F.W., Lei Y.M., Jabri B., Alegre M.L. (2015). Commensal Bifidobacterium promotes antitumor immunity and facilitates anti-PD-L1 efficacy. Science.

[61] Maekawa T., Krauss J.L., Abe T., Jotwani R., Triantafilou M., Triantafilou K., Hashim A., Hoch S., Curtis M.A., Nussbaum G. (2014). Porphyromonas gingivalis manipulates complement and TLR signaling to uncouple bacterial clearance from inflammation and promote dysbiosis. Cell Host Microbe.

[62] Zenobia C., Hajishengallis G. (2015). Porphyromonas gingivalis virulence factors involved in subversion of leukocytes and microbial dysbiosis. Virulence.

[63] Arimatsu K., Yamada H., Miyazawa H., Minagawa T., Nakajima M., Ryder M.I., Gotoh K., Motooka D., Nakamura S., Iida T. (2014). Oral pathobiont induces systemic inflammation and metabolic changes associated with alteration of gut microbiota. Sci. Rep..

[64] Nakajima M., Arimatsu K., Kato T., Matsuda Y., Minagawa T., Takahashi N., Ohno H., Yamazaki K. (2015). Oral Administration of P. gingivalis Induces Dysbiosis of Gut Microbiota and Impaired Barrier Function Leading to Dissemination of Enterobacteria to the Liver. PLoS ONE.

[65] Palm N.W., de Zoete M.R., Cullen T.W., Barry N.A., Stefanowski J., Hao L., Degnan P.H., Hu J., Peter I., Zhang W. (2014). Immunoglobulin A coating identifies colitogenic bacteria in inflammatory bowel disease. Cell.

[66] Hajishengallis G., Liang S., Payne M.A., Hashim A., Jotwani R., Eskan M.A., McIntosh M.L., Alsam A., Kirkwood K.L., Lambris J.D. (2011). Low-abundance biofilm species orchestrates inflammatory periodontal disease through the commensal microbiota and complement. Cell Host Microbe.

[67] Sato K., Takahashi N., Kato T., Matsuda Y., Yokoji M., Yamada M., Nakajima T., Kondo N., Endo N., Yamamoto R. (2017). Aggravation of collagen-induced arthritis by orally administered Porphyromonas gingivalis through modulation of the gut microbiota and gut immune system. Sci. Rep..

[68] Kato T., Yamazaki K., Nakajima M., Date Y., Kikuchi J., Hase K., Ohno H., Yamazaki K. (2018). Oral Administration of Porphyromonas gingivalis Alters the Gut Microbiome and Serum Metabolome. mSphere.

[69] Ottosson F., Brunkwall L., Ericson U., Nilsson P.M., Almgren P., Fernandez C., Melander O., Orho-Melander M. (2018). Connection Between BMI-Related Plasma Metabolite Profile and Gut Microbiota. J. Clin. Endocrinol. Metab..

[70] Konturek P.C., Harsch I.A., Konturek K., Schink M., Konturek T., Neurath M.F., Zopf Y. (2018). Gut(-)Liver Axis: How Do Gut Bacteria Influence the Liver?. Med. Sci..

[71] Qin N., Yang F., Li A., Prifti E., Chen Y., Shao L., Guo J., Le Chatelier E., Yao J., Wu L. (2014). Alterations of the human gut microbiome in liver cirrhosis. Nature.

[72] Merritt M.E., Donaldson J.R. (2009). Effect of bile salts on the DNA and membrane integrity of enteric bacteria. J. Med. Microbiol..

[73] Yoneda M., Naka S., Nakano K., Wada K., Endo H., Mawatari H., Imajo K., Nomura R., Hokamura K., Ono M. (2012). Involvement of a periodontal pathogen, Porphyromonas gingivalis on the pathogenesis of non-alcoholic fatty liver disease. BMC Gastroenterol..

[74] Jiang W., Wu N., Wang X., Chi Y., Zhang Y., Qiu X., Hu Y., Li J., Liu Y. (2015). Dysbiosis gut microbiota associated with inflammation and impaired mucosal immune function in intestine of humans with non-alcoholic fatty liver disease. Sci. Rep..

[75] Komazaki R., Katagiri S., Takahashi H., Maekawa S., Shiba T., Takeuchi Y., Kitajima Y., Ohtsu A., Udagawa S., Sasaki N. (2017). Periodontal pathogenic bacteria, Aggregatibacter actinomycetemcomitans affect non-alcoholic fatty liver disease by altering gut microbiota and glucose metabolism. Sci. Rep..

[76] Zhong Y., Nyman M., Fak F. (2015). Modulation of gut microbiota in rats fed high-fat diets by processing whole-grain barley to barley malt. Mol. Nutr. Food Res..

[77] Lourenςo T.G.B., Spencer S.J., Alm E.J., Colombo A.P.V. (2018). Defining the gut microbiota in individuals with periodontal diseases: An exploratory study. J. Oral Microbiol..

[78] Bajaj J.S., Matin P., White M.B., Fagan A., Deeb J.G., Acharya C., Dalmet S.S., Sikaroodi M., Gillevet P.M., Sahingur S.E. (2018). Periodontal therapy favorably modulates the oral-gut-hepatic axis in cirrhosis. Am. J. Physiol. Gastrointest. Liver Physiol..

[79] Ahn J., Chen C.Y., Hayes R.B. (2012). Oral microbiome and oral and gastrointestinal cancer risk. Cancer Causes Control..

[80] Nakatsu G., Li X., Zhou H., Sheng J., Wong S.H. (2015). Gut mucosal microbiome across stages of colorectal carcinogenesis. Nat. Commun..

[81] Hale V.L., Chen J., Johnson S., Harrington S.C., Yab T.C., Smyrk T.C., Nelson H., Boardman L.A., Druliner B.R., Levin T.R. (2017). Shifts in the Fecal Microbiota Associated with Adenomatous Polyps. Cancer Epidemiol. Biomarkers Prev..

[82] Liang Q., Chiu J., Chen Y., Huang Y., Higashimori A., Fang J., Brim H., Ashktorab H., Ng S.C., Ng S.S.M. (2017). Fecal Bacteria Act as Novel Biomarkers for Noninvasive Diagnosis of Colorectal Cancer. Clin. Cancer Res..

[83] Flemer B., Warren R.D., Barrett M.P., Cisek K., Das A., Jeffery I.B., Hurley E., O’Riordain M., Shanahan F., O’Toole P.W. (2018). The oral microbiota in colorectal cancer is distinctive and predictive. Gut.

[84] Momen-Heravi F., Babic A., Tworoger S.S., Zhang L., Wu K., Smith-Warner S.A., Ogino S., Chan A.T., Meyerhardt J., Giovannucci E. (2017). Periodontal disease, tooth loss and colorectal cancer risk: Results from the Nurses’ Health Study. Int. J. Cancer.

[85] Dejea C.M., Wick E.C., Hechenbleikner E.M., White J.R., Mark Welch J.L., Rossetti B.J., Peterson S.N., Snesrud E.C., Borisy G.G., Lazarev M. (2014). Microbiota organization is a distinct feature of proximal colorectal cancers. Proc. Natl. Acad. Sci. USA.

[86] Yang Y., Cai Q., Shu X.O., Steinwandel M.D., Blot W.J., Zheng W., Long J. (2019). Prospective study of oral microbiome and colorectal cancer risk in low-income and African American populations. Int. J. Cancer.

[87] Kostic A.D., Gevers D., Pedamallu C.S., Michaud M., Duke F., Earl A.M., Ojesina A.I., Jung J., Bass A.J., Tabernero J. (2012). Genomic analysis identifies association of *Fusobacterium* with colorectal carcinoma. Genome Res..

[88] Flanagan L., Schmid J., Ebert M., Soucek P., Kunicka T., Liska V., Bruha J., Neary P., Dezeeuw N., Tommasino M. (2014). Fusobacterium nucleatum associates with stages of colorectal neoplasia development, colorectal cancer and disease outcome. Eur. J. Clin. Microbiol. Infect. Dis.

[89] Signat B., Roques C., Poulet P., Duffaut D. (2011). *Fusobacterium nucleatum* in periodontal health and disease. Curr. Issues Mol. Biol..

[90] Ahn J., Sinha R., Pei Z., Dominianni C., Wu J., Shi J., Goedert J.J., Hayes R.B., Yang L. (2013). Human gut microbiome and risk for colorectal cancer. J. Natl. Cancer Inst..

[91] Rubinstein M.R., Wang X., Liu W., Hao Y., Cai G., Han Y.W. (2013). *Fusobacterium nucleatum* promotes colorectal carcinogenesis by modulating E-cadherin/β-catenin signaling via its FadA adhesin. Cell Host Microbe.

[92] Mima K., Cao Y., Chan A.T., Qian Z.R., Nowak J.A., Masugi Y., Shi Y., Song M., da Silva A., Gu M. (2016). *Fusobacterium nucleatum* in Colorectal Carcinoma Tissue According to Tumor Location. Clin. Transl. Gastroenterol..

[93] Tahara T., Yamamoto E., Suzuki H., Maruyama R., Chung W., Garriga J., Jelinek J., Yamano H.O., Sugai T., An B. (2014). *Fusobacterium* in colonic flora and molecular features of colorectal carcinoma. Cancer Res..

[94] Gao Z., Guo B., Gao R., Zhu Q., Qin H. (2015). Microbiota disbiosis is associated with colorectal cancer. Front. Microbiol..

[95] Sobhani I., Tap J., Roudot-Thoraval F., Roperch J.P., Letulle S., Langella P., Corthier G., Tran Van Nhieu J., Furet J.P. (2011). Microbial dysbiosis in colorectal cancer (CRC) patients. PLoS ONE.

[96] Ito M., Kanno S., Nosho K., Sukawa Y., Mitsuhashi K., Kurihara H., Igarashi H., Takahashi T., Tachibana M., Takahashi H. (2015). Association of *Fusobacterium nucleatum* with clinical and molecular features in colorectal serrated pathway. Int. J. Cancer.

[97] Phipps A.I., Chan A.T., Ogino S. (2013). Anatomic subsite of primary colorectal cancer and subsequent risk and distribution of second cancers. Cancer.

[98] Yamauchi M., Morikawa T., Kuchiba A., Imamura Y., Qian Z.R., Nishihara R., Liao X., Waldron L., Hoshida Y., Huttenhower C. (2012). Assessment of colorectal cancer molecular features along bowel subsites challenges the conception of distinct dichotomy of proximal versus distal colorectum. Gut.

[99] Yamaoka Y., Suehiro Y., Hashimoto S., Hoshida T., Fujimoto M., Watanabe M., Imanaga D., Sakai K., Matsumoto T., Nishioka M. (2018). *Fusobacterium nucleatum* as a prognostic marker of colorectal cancer in a Japanese population. J. Gastroenterol..

[100] Yu T., Guo F., Yu Y., Sun T., Ma D., Han J., Qian Y., Kryczek I., Sun D., Nagarsheth N. (2017). *Fusobacterium nucleatum* Promotes Chemoresistance to Colorectal Cancer by Modulating Autophagy. Cell.

[101] Komiya Y., Shimomura Y., Higurashi T., Sugi Y., Arimoto J., Umezawa S., Uchiyama S., Matsumoto M., Nakajima A. (2019). Patients with colorectal cancer have identical strains of *Fusobacterium nucleatum* in their colorectal cancer and oral cavity. Gut.

[102] Segata N., Haake S.K., Mannon P., Lemon K.P., Waldron L., Gevers D., Huttenhower C., Izard J. (2012). Composition of the adult digestive tract bacterial microbiome based on seven mouth surfaces, tonsils, throat and stool samples. Genome Biol..

[103] Li B., Ge Y., Cheng L., Zeng B., Yu J., Peng X., Zhao J., Li W., Ren B., Li M. (2019). Oral bacteria colonize and compete with gut microbiota in gnotobiotic mice. Int. J. Oral Sci..

[104] Stecher B., Hardt W.D. (2011). Mechanisms controlling pathogen colonization of the gut. Curr. Opin. Microbiol..

[105] Seedorf H., Griffin N.W., Ridaura V.K., Reyes A., Cheng J., Rey F.E., Smith M.I., Simon G.M., Scheffrahn R.H., Woebken D. (2014). Bacteria from diverse habitats colonize and compete in the mouse gut. Cell.

[106] Atarashi K., Suda W., Chen K., Gevers D., Hattori M., Honda K., Johnson R.C., Kiguchi Y., Kolls J.K., Elinav E. (2017). Ectopic colonization of oral bacteria in the intestine drives T(H)1 cell induction and inflammation. Science.

[107] Schmidt T.S., Hayward M.R., Coelho L.P., Li S.S., Costea P.I., Voigt A.Y., Wirbel J., Maistrenko O.M., Alves R.J., Bergsten E. (2019). Extensive transmission of microbes along the gastrointestinal tract. Elife.

[108] Zeller G., Tap J., Voigt A.Y., Sunagawa S., Kultima J.R., Costea P.I., Amiot A., Bohm J., Brunetti F., Habermann N. (2014). Potential of fecal microbiota for early-stage detection of colorectal cancer. Mol. Syst. Biol..

[109] Saygun I., Nizam N., Keskiner I., Bal V., Kubar A., Acikel C., Serdar M., Slots J. (2011). Salivary infectious agents and periodontal disease status. J. Periodontal. Res..

[110] Ridlon J.M., Kang D.J., Hylemon P.B., Bajaj J.S. (2014). Bile acids and the gut microbiome. Curr. Opin. Gastroenterol..

[111] Martinsen T.C., Bergh K., Waldum H.L. (2005). Gastric juice: A barrier against infectious diseases. Basic Clin. Pharmacol. Toxicol..

[112] Walker M.Y., Pratap S., Southerland J.H., Farmer-Dixon C.M., Lakshmyya K., Gangula P.R. (2018). Role of oral and gut microbiome in nitric oxide-mediated colon motility. Nitric. Oxide.

[113] Imhann F., Bonder M.J., Vich Vila A., Fu J., Mujagic Z., Vork L., Tigchelaar E.F., Jankipersadsing S.A., Cenit M.C., Harmsen H.J. (2016). Proton pump inhibitors affect the gut microbiome. Gut.

[114] Geva-Zatorsky N., Sefik E., Kua L., Pasman L., Tan T.G., Ortiz-Lopez A., Yanortsang T.B., Yang L., Jupp R., Mathis D. (2017). Mining the Human Gut Microbiota for Immunomodulatory Organisms. Cell.

[115] Rodriguez Herrero E., Boon N., Pauwels M., Bernaerts K., Slomka V., Quirynen M., Teughels W. (2017). Necrotrophic growth of periodontopathogens is a novel virulence factor in oral biofilms. Sci. Rep..

[116] Parahitiyawa N.B., Jin L.J., Leung W.K., Yam W.C., Samaranayake L.P. (2009). Microbiology of odontogenic bacteremia: Beyond endocarditis. Clin. Microbiol. Rev..

[117] Hajishengallis G. (2015). Periodontitis. From microbial immune subversion to systemic inflammation. Nat. Rev. Immunol..

[118] Tsukasaki M., Komatsu N., Nagashima K., Nitta T., Pluemsakunthai W., Shukunami C., Iwakura Y., Nakashima T., Okamoto K., Takayanagi H. (2018). Host defense against oral microbiota by bone-damaging T cells. Nat. Commun..

[119] Carrion J., Scisci E., Miles B., Sabino G.J., Zeituni A.E., Gu Y., Bear A., Genco C.A., Brown D.L., Cutler C.W. (2012). Microbial carriage state of peripheral blood dendritic cells (DCs) in chronic periodontitis influences DC differentiation, atherogenic potential. J. Immunol..

[120] Kolenbrander P.E., Palmer R.J., Periasamy S., Jakubovics N.S. (2010). Oral multispecies biofilm development and the key role of cell-cell distance. Nat. Rev. Microbiol..

[121] Chenicheri S., Usha R., Ramachandran R., Thomas V., Wood A. (2017). Insight into Oral Biofilm: Primary, Secondary and Residual Caries and Phyto-Challenged Solutions. Open Dent. J..

[122] Li S., Konstantinov S.R., Smits R., Peppelenbosch M.P. (2017). Bacterial Biofilms in Colorectal Cancer Initiation and Progression. Trends Mol. Med..

[123] Warren R.L., Freeman D.J., Pleasance S., Watson P., Moore R.A., Cochrane K., Allen-Vercoe E., Holt R.A. (2013). Co-occurrence of anaerobic bacteria in colorectal carcinomas. Microbiome.

[124] Dejea C.M., Sears C.L. (2016). Do biofilms confer a pro-carcinogenic state?. Gut Microbes.

[125] Drewes J.L., White J.R., Dejea C.M., Fathi P., Iyadorai T., Vadivelu J., Roslani A.C., Wick E.C., Mongodin E.F., Loke M.F. (2017). High-resolution bacterial 16S rRNA gene profile meta-analysis and biofilm status reveal common colorectal cancer consortia. NPJ Biofilms Microbiomes.

[126] Johansson M.E., Hansson G.C. (2016). Immunological aspects of intestinal mucus and mucins. Nat. Rev. Immunol..

[127] Sicard J.F., Le Bihan G., Vogeleer P., Jacques M., Harel J. (2017). Interactions of Intestinal Bacteria with Components of the Intestinal Mucus. Front. Cell Infect. Microbiol..

[128] Jorth P., Turner K.H., Gumus P., Nizam N., Buduneli N., Whiteley M. (2014). Metatranscriptomics of the human oral microbiome during health and disease. MBio.

[129] Hajishengallis G., Lamont R.J. (2012). Beyond the red complex and into more complexity: The polymicrobial synergy and dysbiosis (PSD) model of periodontal disease etiology. Mol. Oral Microbiol..

[130] Gagnière J., Raisch J., Veziant J., Barnich N., Bonnet R., Buc E., Bringer M.A., Pezet D., Bonnet M. (2016). Gut microbiota imbalance and colorectal cancer. World J. Gastroenterol..

[131] Kostic A.D., Chun E., Robertson L., Glickman J.N., Gallini C.A., Michaud M., Clancy T.E., Chung D.C., Lochhead P., Hold G.L. (2013). *Fusobacterium nucleatum* potentiates intestinal tumorigenesis and modulates the tumor-immune microenvironment. Cell Host Microbe.

[132] Amitay E.L., Werner S., Vital M., Pieper D.H., Hofler D., Gierse I.J., Butt J., Balavarca Y., Cuk K., Brenner H. (2017). *Fusobacterium* and colorectal cancer: Causal factor or passenger? Results from a large colorectal cancer screening study. Carcinogenesis.

[133] Tomkovich S., Dejea C.M., Winglee K., Drewes J.L., Chung L., Housseau F., Pope J.L., Gauthier J., Sun X., Muhlbauer M. (2019). Human colon mucosal biofilms from healthy or colon cancer hosts are carcinogenic. J. Clin. Investig..

[134] Bullman S., Pedamallu C.S., Sicinska E., Clancy T.E., Zhang X., Cai D., Neuberg D., Huang K., Guevara F., Nelson T. (2017). Analysis of *Fusobacterium* persistence and antibiotic response in colorectal cancer. Science.

[135] Tomkovich S., Yang Y., Winglee K., Gauthier J., Muhlbauer M., Sun X., Mohamadzadeh M., Liu X., Martin P., Wang G.P. (2017). Locoregional Effects of Microbiota in a Preclinical Model of Colon Carcinogenesis. Cancer Res..

[136] Takahashi N. (2005). Microbial ecosystem in the oral cavity: Metabolic diversity in an ecological niche and its relationship with oral diseases. ICS.

[137] Milella L. (2015). The Negative Effects of Volatile Sulphur Compounds. J. Vet. Dent..

[138] Donaldson G.P., Lee S.M., Mazmanian S.K. (2016). Gut biogeography of the bacterial microbiota. Nat. Rev. Microbiol..

[139] Eley B.M., Cox S.W. (2003). Proteolytic and hydrolytic enzymes from putative periodontal pathogens: Characterization, molecular genetics, effects on host defenses and tissues and detection in gingival crevice fluid. Periodontology 2000.

[140] Linden S.K., Florin T.H., McGuckin M.A. (2008). Mucin dynamics in intestinal bacterial infection. PLoS ONE.

[141] Potempa J., Sroka A., Imamura T., Travis J. (2003). Gingipains, the major cysteine proteinases and virulence factors of Porphyromonas gingivalis: Structure, function and assembly of multidomain protein complexes. Curr. Protein Pept. Sci..

[142] Kilian M., Reinholdt J., Nyvad B., Frandsen E.V., Mikkelsen L. (1989). IgA1 proteases of oral streptococci: Ecological aspects. Immunol. Invest..

[143] Louis P., Hold G.L., Flint H.J. (2014). The gut microbiota, bacterial metabolites and colorectal cancer. Nat. Rev. Microbiol..

[144] Mariggio M.A., Vinella A., Pasquetto N., Curci E., Cassano A., Fumarulo R. (2004). In vitro effects of polyamines on polymorphonuclear cell apoptosis and implications in the pathogenesis of periodontal disease. Immunopharmacol. Immunotoxicol..

[145] Johnson C.H., Dejea C.M., Edler D., Hoang L.T., Santidrian A.F., Felding B.H., Ivanisevic J., Cho K., Wick E.C., Hechenbleikner E.M. (2015). Metabolism links bacterial biofilms and colon carcinogenesis. Cell Metab..

[146] Patel C.N., Wortham B.W., Lines J.L., Fetherston J.D., Perry R.D., Oliveira M.A. (2006). Polyamines are essential for the formation of plague biofilm. J. Bacteriol.

[147] Attene-Ramos M.S., Wagner E.D., Plewa M.J., Gaskins H.R. (2006). Evidence that hydrogen sulfide is a genotoxic agent. Mol. Cancer Res..

[148] Hellmich M.R., Szabo C. (2015). Hydrogen Sulfide and Cancer. Handb. Exp. Pharmacol..

[149] Pavlova S.I., Jin L., Gasparovich S.R., Tao L. (2013). Multiple alcohol dehydrogenases but no functional acetaldehyde dehydrogenase causing excessive acetaldehyde production from ethanol by oral streptococci. Microbiology.

[150] Meurman J.H., Uittamo J. (2008). Oral micro-organisms in the etiology of cancer. Acta. Odontol. Scand..

[151] Muto M., Hitomi Y., Ohtsu A., Shimada H., Kashiwase Y., Sasaki H., Yoshida S., Esumi H. (2000). Acetaldehyde production by non-pathogenic Neisseria in human oral microflora: Implications for carcinogenesis in upper aerodigestive tract. Int. J. Cancer.

[152] Yang L., Ganly I., Morris L., Palmer F., Deng H., Ahn J., Hayes R.B., Wang B., Pei Z. (2011). Relevance of Microbiome to Cigarette Smoking and Oral Cancer. J. Dent. Res..

[153] Verna L., Whysner J., Williams G.M. (1996). N-nitrosodiethylamine mechanistic data and risk assessment: Bioactivation, DNA-adduct formation, mutagenicity, and tumor initiation. Pharmacol. Ther..

[154] Gao J., Xu K., Liu H., Liu G., Bai M., Peng C., Li T., Yin Y. (2018). Impact of the Gut Microbiota on Intestinal Immunity Mediated by Tryptophan Metabolism. Front. Cell Infect. Microbiol..

[155] Lamas B., Richard M.L., Leducq V., Pham H.P., Michel M.L., Da Costa G., Bridonneau C., Jegou S., Hoffmann T.W., Natividad J.M. (2016). CARD9 impacts colitis by altering gut microbiota metabolism of tryptophan into aryl hydrocarbon receptor ligands. Nat. Med..

[156] Rothhammer V., Mascanfroni I.D., Bunse L., Takenaka M.C., Kenison J.E., Mayo L., Chao C.C., Patel B., Yan R., Blain M. (2016). Type I interferons and microbial metabolites of tryptophan modulate astrocyte activity and central nervous system inflammation via the aryl hydrocarbon receptor. Nat. Med..

[157] Smith P.M., Howitt M.R., Panikov N., Michaud M., Gallini C.A., Bohlooly Y.M., Glickman J.N., Garrett W.S. (2013). The microbial metabolites, short-chain fatty acids, regulate colonic Treg cell homeostasis. Science.

[158] Ostaff M.J., Stange E.F., Wehkamp J. (2013). Antimicrobial peptides and gut microbiota in homeostasis and pathology. EMBO Mol. Med..

[159] Rescigno T., Micolucci L., Tecce M.F., Capasso A. (2017). Bioactive Nutrients and Nutrigenomics in Age-Related Diseases. Molecules.

[160] Mehta R.S., Nishihara R., Cao Y., Song M., Mima K., Qian Z.R., Nowak J.A., Kosumi K., Hamada T., Masugi Y. (2017). Association of Dietary Patterns with Risk of Colorectal Cancer Subtypes Classified by *Fusobacterium nucleatum* in Tumor Tissue. JAMA Oncol..

[161] Yu J., Feng Q., Wong S.H., Zhang D., Liang Q.Y., Qin Y., Tang L., Zhao H., Stenvang J., Li Y. (2017). Metagenomic analysis of faecal microbiome as a tool towards targeted non-invasive biomarkers for colorectal cancer. Gut.

[162] Whitmore S.E., Lamont R.J. (2014). Oral bacteria and cancer. PLoS Pathog..

[163] Coppenhagen-Glazer S., Sol A., Abed J., Naor R., Zhang X., Han Y.W., Bachrach G. (2015). Fap2 of *Fusobacterium nucleatum* is a galactose-inhibitable adhesin involved in coaggregation, cell adhesion, and preterm birth. Infect. Immun..

[164] Gur C., Ibrahim Y., Isaacson B., Yamin R., Abed J., Gamliel M., Enk J., Bar-On Y., Stanietsky-Kaynan N., Coppenhagen-Glazer S. (2015). Binding of the Fap2 protein of *Fusobacterium nucleatum* to human inhibitory receptor TIGIT protects tumors from immune cell attack. Immunity.

[165] Abed J., Emgard J.E., Zamir G., Faroja M., Almogy G., Grenov A., Sol A., Naor R., Pikarsky E., Atlan K.A. (2016). Fap2 Mediates *Fusobacterium nucleatum* Colorectal Adenocarcinoma Enrichment by Binding to Tumor-Expressed Gal-GalNAc. Cell Host Microbe.

[166] Han Y.W., Ikegami A., Rajanna C., Kawsar H.I., Zhou Y., Li M., Sojar H.T., Genco R.J., Kuramitsu H.K., Deng C.X. (2005). Identification and characterization of a novel adhesin unique to oral fusobacteria. J. Bacteriol..

[167] Yilmaz O., Yao L., Maeda K., Rose T.M., Lewis E.L., Duman M., Lamont R.J., Ojcius D.M. (2008). ATP scavenging by the intracellular pathogen Porphyromonas gingivalis inhibits P2X7-mediated host-cell apoptosis. Cell Microbiol..

[168] Savio L.E.B., de Andrade Mello P., da Silva C.G., Coutinho-Silva R. (2018). The P2X7 Receptor in Inflammatory Diseases: Angel or Demon?. Front. Pharmacol..

[169] Mao S., Park Y., Hasegawa Y., Tribble G.D., James C.E., Handfield M., Stavropoulos M.F., Yilmaz O., Lamont R.J. (2007). Intrinsic apoptotic pathways of gingival epithelial cells modulated by *Porphyromonas gingivalis*. Cell Microbiol..

[170] Yao L., Jermanus C., Barbetta B., Choi C., Verbeke P., Ojcius D.M., Yilmaz O. (2010). *Porphyromonas gingivalis* infection sequesters pro-apoptotic Bad through Akt in primary gingival epithelial cells. Mol. Oral Microbiol..

[171] Nakhjiri S.F., Park Y., Yilmaz O., Chung W.O., Watanabe K., El-Sabaeny A., Park K., Lamont R.J. (2001). Inhibition of epithelial cell apoptosis by *Porphyromonas gingivalis*. FEMS Microbiol. Lett..

[172] Inaba H., Sugita H., Kuboniwa M., Iwai S., Hamada M., Noda T., Morisaki I., Lamont R.J., Amano A. (2014). Porphyromonas gingivalis promotes invasion of oral squamous cell carcinoma through induction of proMMP9 and its activation. Cell Microbiol..

[173] Thaiss C.A., Levy M., Korem T., Dohnalova L., Shapiro H., Jaitin D.A., David E., Winter D.R., Gury-BenAri M., Tatirovsky E. (2016). Microbiota Diurnal Rhythmicity Programs Host Transcriptome Oscillations. Cell.

[174] Abreu M.T. (2010). Toll-like receptor signalling in the intestinal epithelium: How bacterial recognition shapes intestinal function. Nat. Rev. Immunol..

[175] Underhill D.M., Iliev I.D. (2014). The mycobiota: Interactions between commensal fungi and the host immune system. Nat. Rev. Immunol..

[176] Santaolalla R., Sussman D.A., Ruiz J.R., Davies J.M., Pastorini C., Espana C.L., Sotolongo J., Burlingame O., Bejarano P.A., Philip S. (2013). TLR4 activates the beta-catenin pathway to cause intestinal neoplasia. PLoS ONE.

[177] Chen Y., Peng Y., Yu J., Chen T., Wu Y., Shi L., Li Q., Wu J., Fu X. (2017). Invasive *Fusobacterium nucleatum* activates beta-catenin signaling in colorectal cancer via a TLR4/P-PAK1 cascade. Oncotarget.

[178] Dharmani P., Strauss J., Ambrose C., Allen-Vercoe E., Chadee K. (2011). *Fusobacterium nucleatum* infection of colonic cells stimulates MUC2 mucin and tumor necrosis factor alpha. Infect. Immun..

[179] Ye X., Wang R., Bhattacharya R., Boulbes D.R., Fan F., Xia L., Adoni H., Ajami N.J., Wong M.C., Smith D.P. (2017). *Fusobacterium Nucleatum* Subspecies Animalis Influences Proinflammatory Cytokine Expression and Monocyte Activation in Human Colorectal Tumors. Cancer Prev. Res. (Phila.).

[180] Park H.E., Kim J.H., Cho N.Y., Lee H.S., Kang G.H. (2017). Intratumoral *Fusobacterium nucleatum* abundance correlates with macrophage infiltration and CDKN2A methylation in microsatellite-unstable colorectal carcinoma. Virchows. Arch..

[181] Mima K., Sukawa Y., Nishihara R., Qian Z.R., Yamauchi M., Inamura K., Kim S.A., Masuda A., Nowak J.A., Nosho K. (2015). *Fusobacterium nucleatum* and T Cells in Colorectal Carcinoma. JAMA Oncol..

[182] Sokol H., Mahlaoui N., Aguilar C., Bach P., Join-Lambert O., Garraffo A., Seksik P., Danion F., Jegou S., Straube M. (2019). Intestinal dysbiosis in inflammatory bowel disease associated with primary immunodeficiency. J. Allergy Clin. Immunol..

[183] Kummen M., Holm K., Anmarkrud J.A., Nygard S., Vesterhus M., Hoivik M.L., Troseid M., Marschall H.U., Schrumpf E., Moum B. (2017). The gut microbial profile in patients with primary sclerosing cholangitis is distinct from patients with ulcerative colitis without biliary disease and healthy controls. Gut.

[184] Spooner R., Yilmaz O. (2011). The role of reactive-oxygen-species in microbial persistence and inflammation. Int. J. Mol. Sci..

[185] Choi C.H., Spooner R., DeGuzman J., Koutouzis T., Ojcius D.M., Yilmaz O. (2013). *Porphyromonas gingivalis*-nucleoside-diphosphate-kinase inhibits ATP-induced reactive-oxygen-species via P2X7 receptor/NADPH-oxidase signalling and contributes to persistence. Cell Microbiol.

[186] Szkaradkiewicz A.K., KarpiĹski T.M. (2013). Microbiology of chronic periodontitis. J. Biol. Earth Sci..

[187] Ivanov K., Kolev N., Tonev A., Nikolova G., Krasnaliev I., Softova E., Tonchev A. (2009). Comparative analysis of prognostic significance of molecular markers of apoptosis with clinical stage and tumor differentiation in patients with colorectal cancer: A single institute experience. Hepatogastroenterology.

[188] Guarner F. (2006). Enteric flora in health and disease. Digestion.

